# Weight regain after cessation of medication for weight management: systematic review and meta-analysis

**DOI:** 10.1136/bmj-2025-085304

**Published:** 2026-01-07

**Authors:** Sam West, Jadine Scragg, Paul Aveyard, Jason L Oke, Lia Willis, Stella J P Haffner, Heather Knight, Danni Wang, Sarah Morrow, Laura Heath, Susan A Jebb, Dimitrios A Koutoukidis

**Affiliations:** 1 Nuffield Department of Primary Care Health Sciences, University of Oxford, Oxford OX2 6GG, UK; 2 National Institute for Health Research Oxford Biomedical Research Centre, Oxford University Hospitals NHS Foundation Trust, Oxford, UK; 3 National Institute for Health Research, Oxford Health Biomedical Centre, Warneford Hospital, Oxford, UK; 4 National Institute for Health Research Applied Research Collaboration, Oxford University Hospitals NHS Foundation Trust, Oxford, UK

## Abstract

**Objectives:**

To quantify and compare the rate of weight regain after cessation of weight management medications (WMMs) in adults with overweight or obesity.

**Design:**

Systematic review and meta-analysis.

**Study selection:**

Trial registries and databases (Medline, Embase, PsycINFO, CINAHL, Cochrane, Web of Science, and trial registries) were searched from inception until February 2025 for randomised controlled trials (RCTs), non-randomised trials, and observational studies that included WMM (≥8 weeks) with follow-up for ≥4 weeks after cessation of treatment in adults with overweight or obesity. Comparators were any non-drug weight loss intervention or placebo.

**Data extraction and synthesis:**

The review followed the Preferred Reporting Items for Systematic Reviews and Meta-analyses guidelines. Two independent reviewers screened titles, extracted data, and assessed the risk of bias using the Cochrane Risk of Bias 2 tool for RCTs and the ROBINS-I tool for non-randomised trials. Data were analysed using mixed effect, meta-regression, and time-to-event models. Weight regain after cessation of WMM was compared with that reported after cessation of behavioural weight management programmes (BWMPs).

**Main outcome measures:**

The primary outcome was rate of weight regain from end of treatment, with associated changes in cardiometabolic markers as a secondary outcome.

**Results:**

Of the 9288 titles screened, 37 studies (63 intervention arms, 9341 participants) were included. Average treatment duration was 39 (range 11-176) weeks, with average follow-up of 32 (4-104) weeks. The average monthly rate of weight regain was 0.4 kg (95% confidence interval (CI) 0.3 to 0.5) (mixed model 0.3 kg (0.2 to 0.4) monthly *v* control in RCTs). All cardiometabolic markers were projected to return to baseline within 1.4 years after the cessation of WMM. Weight regain was faster after WMM than after BWMP (by 0.3 kg (0.22 to 0.34) monthly), independent of initial weight loss. Estimates and precision were robust in sensitivity analyses.

**Conclusions:**

This review found that cessation of WMM is followed by rapid weight regain and reversal of beneficial effects on cardiometabolic markers. Regain after WMM was faster than after BWMP. These findings suggest caution in short term use of these drugs without a more comprehensive approach to weight management.

**Systematic review registration:**

PROSPERO CRD42024532069.

**Figure:**
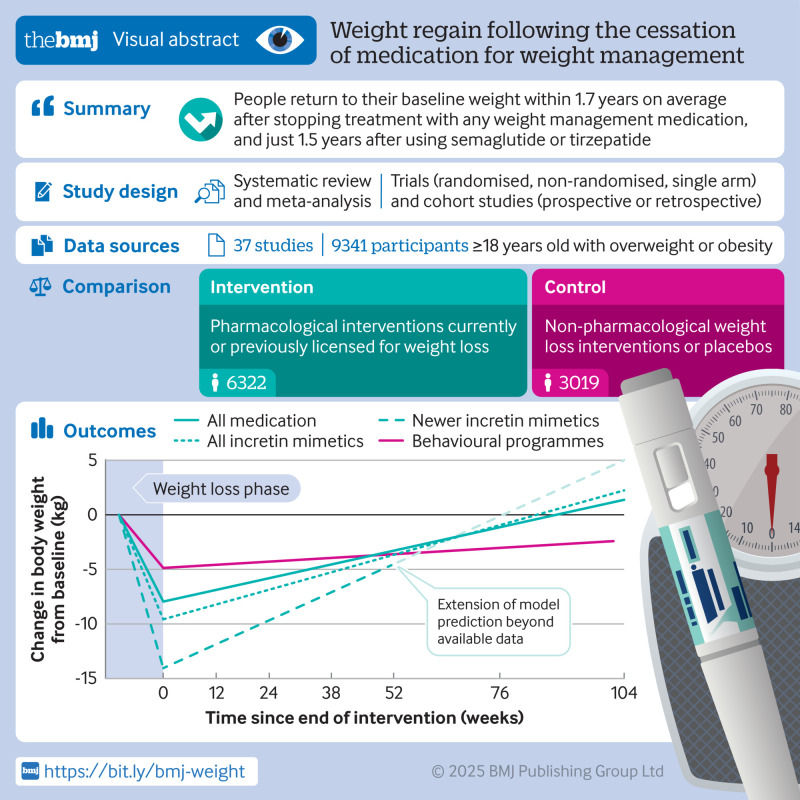


## Introduction

Obesity, a chronic and relapsing condition, affects almost two billion adults worldwide^1^ and increases the risk of morbidity and premature mortality.^2^ ^3^ Behavioural weight management programmes (BWMPs) that provide support to adopt a low energy diet and increased physical activity are the cornerstone of obesity management.^4^ However, new drugs, including the glucagon-like peptide-1 (GLP-1) receptor agonist semaglutide and GLP-1 receptor agonist and glucose-dependent insulinotropic polypeptide (GIP) dual agonist tirzepatide, are set to transform routine obesity treatment, with participants in clinical trials losing 15-20% of their baseline body weight.^5^ ^6^ ^7^ Regardless of the type of treatment, improvements in cardiometabolic markers and endpoints are broadly proportional to the magnitude of weight lost, and these new weight management medications (WMMs) have been shown to lead to notable short term improvements in liver fibrosis, renal outcomes, and sleep apnoea^8^ and in reduced incidence of cardiovascular disease.^9^

In a previous systematic review, we compared weight change after the withdrawal of BWMPs with weight change after either less intensive support or no support. After a mean 2.4 kg greater initial weight loss with BWMPs, weight regain was 0.02 kg per month compared with control over a 10 year follow-up. Initial improvements in cardiometabolic markers attenuated over time but with evidence of benefit for at least five years.^10^ ^11^ ^12^ With the continued development of new incretin mimetic related treatments,^13^ the use of WMMs is likely to become more common. Real world observations estimate that 50% of people with obesity discontinue GLP-1 receptor agonists within 12 months of initiation,^14^ ^15^ so it is important to characterise what happens to body weight after cessation of treatment.

Following the method of our previous review of BWMPs, we quantified the rate of weight regain after the cessation of any WMM and the associated changes in cardiometabolic health markers. Given the greater effectiveness and increasing use of newer incretin mimetic treatment, we specifically examined the rate of weight regain after use of these drugs. Lastly, we examined the rate of weight regain after WMM compared with BWMPs.

## Methods

The protocol for this systematic review and meta-analysis was prospectively registered with PROSPERO and the paper is reported according to the Preferred Reporting Items for Systematic Reviews and Meta-analyses guidelines.^16^ We used data from a previous prospectively registered systematic review and meta-analysis^12^ to compare outcomes after the cessation of interventions using WMM and BWMPs. Details of this review can be found in previous publications,^10^ ^11^ ^12^ and supplementary table 5 provides comparisons of the population, intervention, comparison, outcome, and study type for each review. Briefly, the BWMP review included only randomised controlled trials of behavioural interventions of dietary and physical activity support compared with either no intervention or less intensive support in adults (≥18 years) with overweight or obesity at study start (body mass index ≥25 or ≥23 in Asian populations). To be included, studies had to follow up participants for ≥12 months from baseline and include a measure of weight change at intervention end and after further follow-up without intervention.

The present review included randomised controlled trials and non-randomised comparative trials in which participants were allocated to use of or no use of WMM and were otherwise treated similarly, of adults (≥18 years) with overweight or obesity at the start of the intervention (following the definitions of overweight or obesity outlined in each individual study). In addition, this review also included single arm trials, and prospective and retrospective observational cohorts to maximise data availability, recognising that fewer trials of these drugs exist than for BWMPs.

We included studies using any drug intervention that is currently or has previously been licensed for weight loss, or where there is reason to believe that the drug studied shares a class effect with a currently or previously licensed drug. Drugs included semaglutide, tirzepatide, liraglutide, exenatide, cagrilintide, orlistat, phentermine+topiramate, lorcaserin, naltrexone+bupropion, sibutramine, rimonabant, phentermine, topiramate, benzphetamine, diethylpropion hydrochloride, phendimetrazine, fenfluramine, and dexfenfluramine. WMM treatment had to last for ≥8 weeks, with a follow-up of ≥4 weeks after the cessation of treatment. Studies could combine drug and behavioural interventions. Comparators were not applicable for single arm trials. For controlled trials, the comparator could include any non-drug intervention, such as behavioural interventions, placebo, or no support. To be included in the randomised controlled trial analysis, studies had to have a control group for both the treatment and the post-treatment follow-up period. The comparison period started when both intervention and comparator ceased and the interventions applied to each, if any, were otherwise similar.

We systematically searched Medline, Embase, PsycINFO, CINAHL, Cochrane, Web of Science, and trial registries until February 2025. An experienced librarian created the search strategy (see supplementary table 1). We also hand searched the reference lists of included studies and systematic reviews for potentially relevant articles. No restrictions applied on language or publication date.

Data were analysed in R v4.3.3. The primary outcome was summary estimates of weight change after the cessation of WMM. Two reviewers used the Covidence tool to independently screen studies, and data were extracted independently in duplicate using a predefined data extraction form (SW, LW, JS, SH, HK, DW, SM, LH, DAK). Risk of bias was assessed independently in duplicate. We used the Cochrane Risk of Bias 2 tool for randomised controlled trials and the ROBINS-I (Risk Of Bias In Non-randomised Studies-of Interventions) tool for all other trials. Discrepancies were resolved through discussion or referral to a third reviewer. Study authors were contacted for additional data when required. We assessed publication bias with funnel plots and Egger’s test.^17^ The GRADE (Grading of Recommendations Assessment, Development and Evaluation) approach was used to assess the certainty of evidence.^18^

In line with the BWMP review,^12^ we analysed the data using three different approaches: a mixed model (model 1) with a random intercept for each study, regressing outcomes against all time points since the cessation of the WMM; a meta-regression model (model 2), assuming linear increases in outcomes plotted as baseline and value at longest follow-up; and time-to-event model (model 3), evaluating the time at which the outcome returned to no difference (ie, to baseline weight for the analysis of all studies or to no difference between intervention and control for the randomised controlled trial analysis).

In our primary analysis, we calculated the absolute rate of weight regain after the cessation of WMM. Prespecified subgroup analyses included comparison between different WMM classes (all WMM, all incretin mimetics, and newer more effective incretin mimetics (semaglutide and tirzepatide)), and comparison with our previous review assessing the rate of weight regain after BWMPs.^12^ As secondary analysis, we calculated the change in cardiometabolic markers (HbA_1c_, fasting glucose, systolic blood pressure, diastolic blood pressure, total cholesterol, and triglycerides) after the cessation of WMM.

Weight regain data are expressed as weight change from baseline (pre-intervention) or difference in weight change from baseline between intervention and control for randomised controlled trials. When analysing and presenting data from all studies, we used weight change from single arm trials, observational studies, and the intervention groups from randomised controlled trials. When analysing data from randomised controlled trials only, we calculated the difference in weight change between the intervention and control groups at the end of the intervention and at each available time point after the end of the intervention. When studies had multiple intervention arms, we treated each arm as a separate arm and divided the number in the comparator by the number of intervention arms to avoid duplicative counting.^19^ The models returned weekly estimates, which have been converted to monthly rates of regain for ease of interpretation.

A prespecified sensitivity analysis was performed including only studies with a low risk of bias. We also assessed whether the rate of weight regain varied depending on the type of treatment provided during the follow-up period with no treatment, comparing behavioural support with no support and active treatment (behavioural support or metformin) with non-active treatment (placebo or nothing). In addition to the linear models, following peer review and acknowledging that weight regain might not be linear, we ran the models with a curvilinear fit.

Comparisons with the meta-analysis of BWMPs focused on the intervention groups from each review only. This is because, in some cases, the control group in the WMM review would be considered an intervention group in the BWMP review. To ensure comparability between reviews we recalculated weight regain after the end of BWMP, limiting follow-up to two years as this was the longest available follow-up data across WMM studies. To control for differences in weight loss between WMM and BWMP, we ran a mixed model with weight change, intervention, and initial weight loss as a three-way interaction term to investigate whether differences in initial weight loss would explain differences in the rate of weight regain.

### Patient and public involvement

A standing panel of people advise on our weight management research. They have expressed general concern about weight regain after use of weight loss drugs, although they did not contribute directly to this analysis. We have invited people with experience of weight loss drugs to help develop our dissemination plan.

## Results

Excluding duplicates, 9288 titles and abstracts were screened and 228 full texts assessed (see supplementary figure 1). In alphabetical order of the first author, both table 1 and table 2 detail the characteristics of the intervention, comparator, and follow-up treatment for each study included in the review (also see supplementary table 2). Thirty seven studies (63 intervention arms, 9341 participants) were included in the analysis. Thirty five of the included studies were randomised controlled trials—however, only 28 of these studies had a control group during both intervention and follow-up and were included in the analysis of randomised controlled trials. The randomised controlled trials that did not have a control group during both intervention and follow-up were handled as single arm trials in the analysis.

**Table 1:** Characteristics of studies included in analysis, alphabetised by first author (A to L). All studies were randomised controlled trials unless stated otherwise

Author	Intervention				Comparator				Follow-up	
	No	Details	Duration (weeks)		No	Details	Duration (weeks)		Details	Duration (weeks)
Aronne 2023^7^*	335	Tirzepatide 15 mg/wk	36		-	-	-		Placebo	52
Brownell 1981^20^	69	Fenfluramine 160 mg/d	16		43	Behavioural	16		Behavioural	52
Craighead 1984^21^	16	Fenfluramine 160 mg/d	16		16	Behavioural	16		Nothing	52
Craighead 1984^21^	14	Fenfluramine 160 mg/d	16		16	Behavioural	16		Nothing	52
Craighead 1984^21^	13	Fenfluramine 160 mg/d	8		16	Behavioural	16		Nothing	60
Craighead 1984^21^	15	Fenfluramine 160 mg/d	8		16	Behavioural	16		Nothing	52
Craighead 1981^22^	26	Fenfluramine 120 mg/d	26		34	Behavioural	26		Behavioural	52
Craighead 1981^22^	33	Fenfluramine 120 mg/d	26		34	Behavioural	26		Behavioural	52
Croghan 2016^23^	14	Lorcaserin 20 mg/d	12		16	Low level laser therapy	12		Nothing	12
Croghan 2016^23^	15	Lorcaserin 20 mg/d	12		16	Low level laser therapy	12		Nothing	12
Davidson 1999^24^	141	Orlistat 360 mg/d	52		133	Placebo	52		Placebo	52
Davies 2015^25^	317	Liraglutide 3.0 mg/d	56		116	Placebo	56		Nothing	12
Davies 2015^25^	157	Liraglutide 1.8 mg/d	56		116	Placebo	56		Nothing	12
Dawson 2011^26^	8	Rimonabant 20 mg/d	52		8	Placebo	52		Metformin	12
Early 2007^27^*	49	Sibutramine 15 mg/d	12		-	-	-		Behavioural	36
Ferjan 2017^28^*	12	Liraglutide 3.0 mg/d	12		-	-	-		Metformin	12
Ferjan 2017^28^*	12	Liraglutide 3.0 mg/d	12		-	-	-		Metformin+sitagliptin	12
Grilo 2014^29^	22	Sibutramine 15 mg/d	16		22	Placebo	16		Nothing	52
Grilo 2014^29^	20	Sibutramine 15 mg/d	16		22	Placebo	16		Nothing	52
Jastreboff 2024^30^	172	Tirzepatide 5.0 mg/wk	176		131	Placebo	72		Nothing	17
Jastreboff 2024^30^	185	Tirzepatide 10 mg/wk	176		131	Placebo	72		Nothing	17
Jastreboff 2024^30^	184	Tirzepatide 15 mg/wk	176		131	Placebo	72		Nothing	17
Jensterle 2024^31^†	25	Semaglutide 1 mg/wk	16		-	-	-		Metformin	104
Karhunen 2000^32^	17	Orlistat 360 mg/d	52		19	Placebo	52		Placebo	52
Khoo 2019^33^	15	Liraglutide 3.0 mg/d	26		15	Behavioural	26		Behavioural	26
Kwon 2022^34^*	24	Orlistat	12		-	-	-		Nothing	24
Kwon 2022^34^*	27	Phentermine 37.5 mg/d	12		-	-	-		Nothing	24
Lau 2021^35^	101	Cagrilintide 0.3 mg/wk	26		101	Placebo	26		Nothing	6
Lau 2021^35^	100	Cagrilintide 0.6 mg/wk	26		101	Placebo	26		Nothing	6
Lau 2021^35^	102	Cagrilintide 1.2 mg/wk	26		101	Placebo	26		Nothing	6
Lau 2021^35^	102	Cagrilintide 2.4 mg/wk	26		101	Placebo	26		Nothing	6
Lau 2021^35^	101	Cagrilintide 4.5 mg/wk	26		101	Placebo	26		Nothing	6
Lau 2021^35^	99	Liraglutide 3.0 mg/d	26		101	Placebo	26		Nothing	6
leRoux 2017^45^	783	Liraglutide 3.0 mg/d	160		326	Placebo	160		Behavioural	12
Liang 2014^46^‡	9	Topiramate 50 mg/d	16		-	-	-		Nothing	52

* Not included in randomised controlled trial analysis as these studies were randomised controlled trials by design but did not have a placebo group during treatment and off-treatment follow-up phases.

† Observational study.

‡ Single arm trial.

**Table 2:** Characteristics of randomised controlled trials included in analysis, alphabetised by first author (M to W)

Author	Intervention				Comparator				Follow-up	
	No	Details	Duration (weeks)		No	Details	Duration (weeks)		Details	Duration (weeks)
Marbury 1996^47^	54	Dexfenfluramine 10 mg/d	12		54	Behavioural	12		Behavioural	4
Marbury 1996^47^	57	Dexfenfluramine 30 mg/d	12		54	Behavioural	12		Behavioural	4
Marbury 1996^47^	59	Dexfenfluramine 60 mg/d	12		54	Behavioural	12		Behavioural	4
McGowan 2024^48^	138	Semaglutide 2.4 mg/wk	52		69	Placebo	52		Behavioural	28
Moolla 2025^37^	15	Liraglutide 1.8 mg/d	12		14	Behavioural	12		Nothing	12
Napolitano 2012^49^	19	Sibutramine 10 mg/d	12		19	Placebo	12		Nothing	12
Oneil 2018^50^	92	Semaglutide 0.05 mg/wk	52		123	Placebo	52		Nothing	7
Oneil 2018^50^	96	Semaglutide 0.4 mg/wk	52		123	Placebo	52		Nothing	7
Oneil 2018^50^	100	Semaglutide 0.3 mg/wk FE	52		123	Placebo	52		Nothing	7
Oneil 2018^50^	100	Semaglutide 0.4 mg/wk FE	52		123	Placebo	52		Nothing	7
Oneil 2018^50^	96	Liraglutide 3.0 mg/d	52		123	Placebo	52		Nothing	7
Pi-Sunyer 2006^51^	323	Rimonabant 20 mg/d	52		292	Placebo	52		Placebo	52
Pi-Sunyer 2015^52^	350	Liraglutide 3.0 mg/d	56		304	Placebo	56		Behavioural	12
Rodin 1988^53^	16	Diethylpropion hydrochloride 75 mg/d	11		16	Placebo	11		Nothing	32
Rosenstock 2023^54^	98	Tirzepatide 5.0 mg/wk	40		57	Placebo	40		Nothing	4
Rosenstock 2023^54^	90	Tirzepatide 10 mg/wk	40		57	Placebo	40		Nothing	4
Rosenstock 2023^54^	78	Tirzepatide 15 mg/wk	40		57	Placebo	40		Nothing	4
Rubino 2021^5^*	268	Semaglutide 2.4 mg/wk	20		-	-	-		Behavioural	48
Samp 2015^55^*	18	Orlistat 360 mg/d	36		-	-	-		Nothing	12
Samp 2015^55^*	18	Sibutramine 15 mg/d	36		-	-	-		Nothing	12
Sathyapalan 2009^56^	10	Rimonabant 20 mg/d	12		10	Metformin	12		Metformin	12
Sjostrom 1998^57^	138	Orlistat 360 mg/d	52		123	Placebo	52		Placebo	52
Smith 2010^58^	266	Lorcaserin 20 mg/d	52		665	Placebo	52		Behavioural	52
Svensson 2019^59^	47	Liraglutide 1.8 mg/d	16		50	Placebo	16		Nothing	52
Wadden 2013^60^	159	Liraglutide 3 mg/d	56		144	Placebo	56		Nothing	12
Wilding 2022^6^	228	Semaglutide 2.4 mg/wk	68		99	Placebo	68		Nothing	52
Woo 2007^36^*	28	Orlistat 360 mg/d	26		-	-	-		Nothing	26
Woo 2007^36^*	27	Orlistat 360 mg/d	26		-	-	-		Behavioural	26

FE=fast escalation.

* Not included in randomised controlled trial analysis as these studies were randomised controlled trials by design but did not have a placebo group during treatment and off-treatment follow-up phases.

In most studies, the support provided after the WMM ended was identical except for two studies, where authors compared metformin with no metformin^28^ and behavioural support with no behavioural support.^36^ Sensitivity analysis showed that the rate of weight regain did not differ depending on the type of support provided during the follow-up phase, and therefore we handled all studies similarly in the main analysis (see supplementary figures 4 and 5). Adding a term to allow a curvilinear model did not improve the fit nor differ from linear models (see supplementary figures 8-10).

The number of treatment arms for each WMM was: semaglutide (n=8), tirzepatide (n=7), liraglutide (n=12), cagrilintide (n=5), orlistat (n=7), phentermine (n=2), fenfluramine (n=7), dexfenfluramine (n=3), rimonabant (n=3), sibutramine (n=5), diethylpriopion hydrochloride (n=1), lorcaserin (n=3), and topiramate (n=1). The mean duration of treatment with WMMs was 39 (range 11-176) weeks with a post-WMM follow-up of 32 (4-104) weeks. Only one study included follow-up beyond one year.^31^ The mean weight loss in the WMM groups during the active weight loss phase was 8.3 kg (95% CI 7.2 to 9.5) compared with 3.2 kg (2.5 to 3.9) in the control groups.

**Fig 1:**
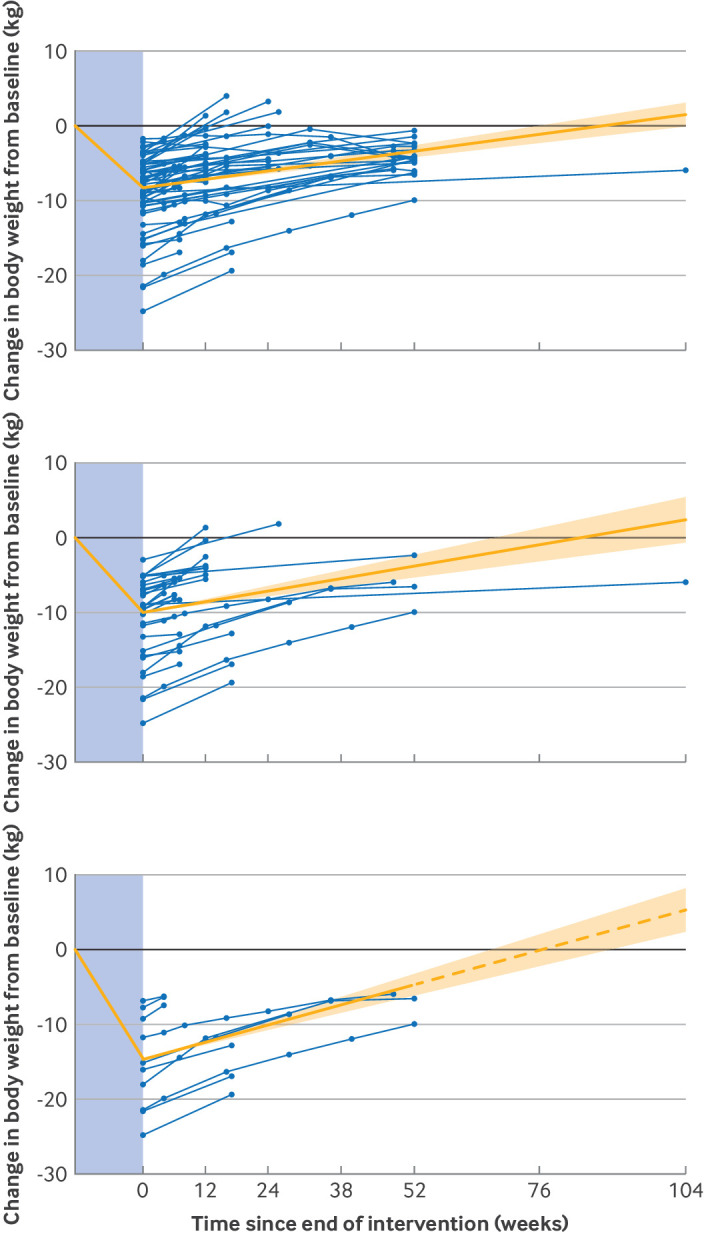
Change in body weight (kg) from baseline (treatment start) for all studies using all weight management medications (top panel), all incretin mimetics (middle panel), and newer and more effective incretin mimetics (semaglutide and tirzepatide) (bottom panel). Solid orange line shows the amount of weight loss during treatment (in the blue shaded area) followed by the rate of weight regain after treatment end (95% confidence interval) as estimated by mixed effects model (model 1); dashed orange line is an extension of the model prediction line to depict the length of time for body weight to return to baseline, as follow-up data were not available beyond 52 weeks for newer and more effective incretin mimetics; and blue lines display individual studies included in the analysis

Figure 1 displays the rate of weight regain from all studies for all WMMs and for the subgroups of all incretin mimetics and newer and more effective incretin mimetics. On average, weight loss at cessation of treatment was 8.3 kg (7.2 to 9.5), 10.1 kg (8.2 to 11.9), and 14.7 kg (11.1 to 18.4), respectively. The mixed model (model 1) estimated a monthly rate of weight regain of 0.4 kg (95% CI 0.3 to 0.5), 0.5 kg (0.4 to 0.7), and 0.8 kg (0.7 to 0.9) for any WMM (63 intervention arms, 6322 participants), all incretin mimetics (32 intervention arms, 4757 participants), and newer and more effective incretin mimetics (10 intervention arms, 1776 participants), respectively. The estimated weight regain was 4.8 kg (3.6 to 6.0), 6.0 kg (4.8 to 8.4), and 9.9 kg (8.4 to 10.8) within the first year after stopping treatment, and a projected return to baseline weight (model 3) by 1.7 years (95% CI 1.3 to 2.1), 1.6 years (1.1 to 2.1), and 1.5 years (1.0 to 1.9) after cessation of any WMM, incretin mimetics, and newer and more effective incretin mimetics, respectively. The evidence for rate of weight regain was judged to be moderate certainty.

**Fig 2:**
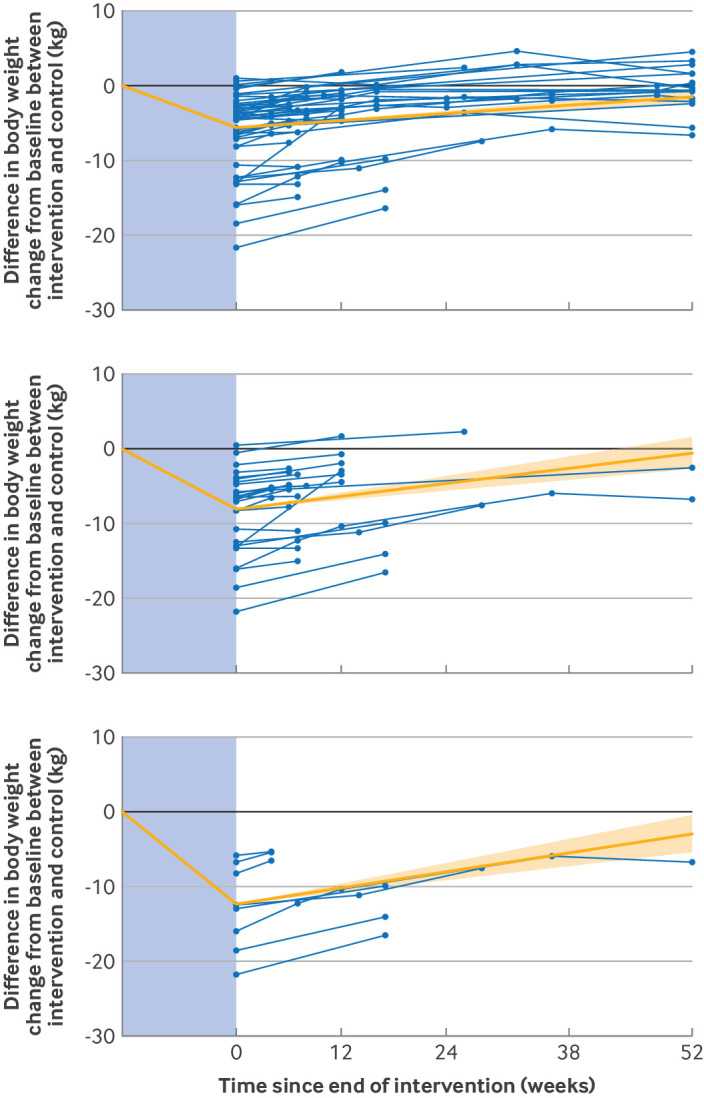
Difference in change in body weight (kg) from baseline (treatment start) between intervention and control for randomised controlled trials using all weight management medications (top panel), all incretin mimetics (middle panel), and newer and more effective incretin mimetics (semaglutide and tirzepatide) (bottom panel). Solid orange line shows the amount of weight loss during treatment (in the blue shaded area) followed by the rate of weight regain after treatment end (95% confidence interval) compared with control, estimated by mixed effects model (model 1); and blue lines display change in body weight in intervention group relative to control for studies included in the analysis

Figure 2 displays the difference in weight regain between groups previously treated with WMM compared with no treatment (control) from the randomised controlled trials only, for all WMMs, incretin mimetics, and newer and more effective incretin mimetics. From the mixed model (model 1), weight loss was 5.7 kg (95% CI 4.4 to 6.9), 8.0 kg (6.1 to 9.9), and 12.3 kg (8.6 to 15.9) compared with control for all WMMs (50 intervention arms), incretin mimetics (27 intervention arms), and newer and more effective incretin mimetics (eight intervention arms), respectively. This model estimated a significantly higher rate of monthly weight regain compared with control of 0.3 kg (95% CI 0.3 to 0.4) for all WMMs (P<0.001), 0.6 kg (0.4 to 0.8) for incretin mimetics (P<0.001), and 0.8 kg (0.6 to 0.9) for newer and more effective incretin mimetics (P<0.001). The time-to-event model (model 3) estimated that the time to no difference between intervention and control was 1.4 years (95% CI 0.9 to 1.8), 1.1 years (0.6 to 1.5), and 1.3 years (0.8 to 1.8) after cessation of WMMs, incretin mimetics, and newer and more effective incretin mimetics, respectively.

Twenty eight studies were included in the meta-regression (model 2) comparing weight change after cessation of WMM to control in the randomised controlled trials (see supplementary figure 2). This model provided a more modest estimate of weight loss during the active phase (6.2 kg, 95% CI 4.7 to 7.8) and monthly rate of weight regain (0.4 kg, 95% CI 0.2 to 0.7), with an estimated time to no difference (model 3) between intervention and control of 1.2 years (95% CI 0.9 to 2.1). This estimate was consistent with the estimate from the mixed model but with less precision. Data were insufficient to perform the meta-regression for incretin mimetics and newer and more effective incretin mimetics.

**Fig 3:**
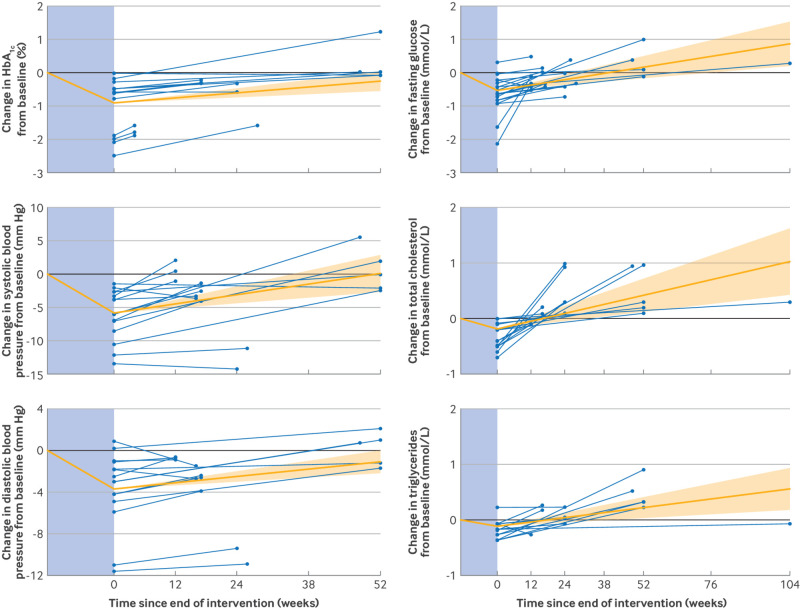
Change in cardiometabolic markers from baseline (treatment start) for all studies included in the review. Solid orange line shows change during treatment (in the blue shaded area) and rate of change after treatment end (95% confidence intervals) as estimated by mixed effects model (model 1); and blue lines display individual studies included in the analysis

Figure 3 displays the changes in cardiometabolic markers after cessation of WMM for all studies analysed using a mixed model (model 1). Fourteen studies reported glycated haemoglobin (HbA_1c_) levels after cessation of WMM. In people taking WMMs, HbA_1c_ levels decreased by 0.9 mmol/mol (95% CI 0.5 to 1.3) during active treatment, then increased at a monthly rate of 0.05 mmol/mol (95% CI 0.03 to 0.08) after treatment cessation. Twenty one studies reported fasting glucose levels after cessation of WMM. By the end of treatment with WMMs, fasting glucose levels had decreased by 0.5 mmol/L (95% CI 0.3 to 0.7) and then increased at a monthly rate of 0.06 mmol/L (95% CI 0.03 to 0.08) after treatment cessation.

Fifteen studies reported changes in systolic and diastolic blood pressure after WMM. Active treatment decreased systolic blood pressure by 5.8 mm Hg (95% CI 3.6 to 7.9), which then increased at a monthly rate of 0.5 mm Hg (95% CI 0.3 to 0.7) after treatment cessation. Diastolic blood pressure decreased by 3.7 mm Hg (1.9 to 5.5) by the end of active treatment with WMMs and then increased at a monthly rate of 0.2 mm Hg (0.1 to 0.3).

Fourteen studies reported changes in total cholesterol concentration after WMM. Cholesterol concentrations decreased by 0.2 mmol/L (95% CI 0.002 to 0.4) and then increased at a monthly rate of 0.05 mmol/L (95% CI 0.03 to 0.07). Fourteen studies reported changes in circulating triglyceride concentrations after WMM. Triglyceride concentrations decreased by 0.2 mmol/L (0.03 to 0.3) and then increased at a monthly rate of 0.03 mmol/L (0.01 to 0.04). In the time-to-event analysis, fasting glucose, systolic blood pressure, and total cholesterol and triglycerides were projected to return to baseline levels within a year after cessation of WMM, with both HbA_1c_ and diastolic blood pressure predicted to return to baseline levels within 1.4 years (95% CI 0.4 to 2.4).

**Fig 4:**
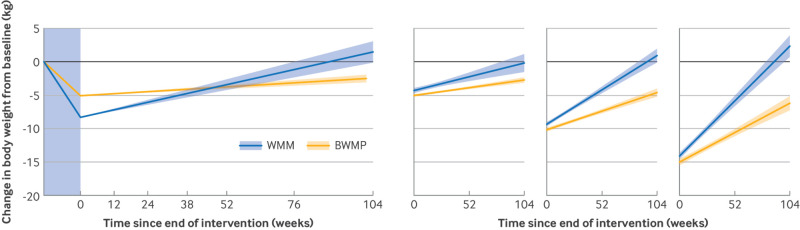
Change in body weight (kg) from baseline (treatment start) (left panel) and for a fixed amount (5 kg, 10 kg, and 15 kg) of weight loss (right panel) after treatment with WMM or BWMPs. Solid lines show weight loss during treatment (in the blue shaded area) and rate of weight regain after treatment end (95% confidence interval) estimated using mixed effects model (model 1). BWMP=behavioural weight management programme; WMM=weight management medication

Figure 4 compares weight regain after WMM with a reanalysis of data from our previous review of BWMPs assessing weight change to two years post-treatment. Weight loss at the end of BWMPs was 5.1 kg (95% CI 4.6 to 5.6) with an estimated monthly weight regain of 0.1 kg (95% CI 0.08 to 0.13). On average, weight loss with WMM was 3.2 kg (95% CI 2.1 to 4.3; P<0.001) greater than that with BWMPs, but monthly weight regain was significantly faster after WMM than after BWMPs by 0.3 kg (95% CI 0.22 to 0.34; P<0.001). Body weight after BWMPs was predicted to return to baseline 3.9 years (95% CI 2.8 to 4.9) after the end of treatment, compared with 1.7 years (1.3 to 2.1) after WMM. In a sensitivity analysis based on a fixed initial weight loss of 5 kg, 10 kg, and 15 kg, larger initial weight loss led to faster weight regain for both WMM and BWMPs, and the rate of regain was consistently faster after WMM than after BWMPs (P<0.001) (fig 4).

We found no evidence that the intensity of behavioural support provided during treatment with WMM impacted the rate of weight regain after treatment (see supplementary figure 3). Nor did we find evidence that behavioural weight management support compared with no support or provision of any intervention (behavioural/metformin versus placebo/nothing) after cessation of WMM altered rates of weight regain (see supplementary figures 4 and 5). In studies using incretin mimetics only, weight loss was 6.7 kg (95% CI 2.7 to 10.5) greater when behavioural support was provided during active treatment, and monthly weight regain of 0.3 kg (95% CI 0.2 to 0.4) was faster (see supplementary figure 3) compared with little or no support. After adjusting for the length of treatment with incretin mimetics, weight loss was still 4.6 kg (95% CI 0.9 to 8.4) greater when behavioural support was provided. After adjusting for initial weight loss, the rate of weight regain did not differ depending on the intensity of behavioural support.

Supplementary table 3 presents a summary of the risk of bias assessments. Twelve randomised controlled trials were judged to have a low risk of bias across all domains, five studies were judged to have some concerns, and 18 studies were judged to have a high risk of bias. Of the studies assessed using the ROBINS-I tool, one was judged to have a serious risk of bias and one was judged to have a critical risk of bias. On visual inspection, little evidence of publication bias was found in the randomised controlled trials (see supplementary figure 6) and trials analysed as single arm trials (see supplementary figure 7) consistent with Egger’s test (P>0.05). In sensitivity analysis including only studies with low risk of bias, monthly weight regain was faster (0.65 kg, 95% CI 0.52 to 0.74) when data for all WMMs was analysed from all studies. Analysis of only studies at low risk of bias showed that the results for weight regain using all incretin mimetics and newer more effective incretin mimetics only were similar to the main analysis (see supplementary table 4).

## Discussion

In this systematic review of 37 studies on weight change after cessation of any WMM, weight regain occurred at an average monthly rate of 0.4 kg, projecting a return to baseline weight 1.7 years after cessation of WMM. Among 28 randomised controlled trials, weight was no different between treatment and control groups 1.4 years after cessation of WMM. Cardiometabolic markers (HbA_1c_, fasting glucose, cholesterol, triglycerides, systolic and diastolic blood pressure) were estimated to be close to baseline by the end of observed follow-up, and extrapolation suggests that they would return to baseline levels within 1.4 years of cessation of WMM. Indirect comparisons showed that people regained weight faster after stopping WMM compared with BWMP, regardless of how much weight was lost during treatment.

### Strengths and limitations of this review

This review was prospectively registered and followed the Cochrane procedures. We used three methods of analysis (mixed model, meta-regression, and time-to-event models) and all provided similar results, adding certainty to our findings. We identified just one study with follow-up of two years after cessation of semaglutide (1 mg) treatment. Data for the newer and more effective incretin mimetics, which are likely to become the dominant treatments, were limited to only eight studies with a maximum of 12 months’ follow-up after cessation of WMM. Data were projected for this category beyond this time point. Furthermore, the time-to-event models were based on linear trajectories for weight regain. Although weight regain may not always be linear, our sensitivity analysis found no evidence of departure from linearity. The previous search of BWMPs was not updated, but the analysis is based on a large body of evidence from 249 randomised controlled trials of BWMPs with 43 151 participants. We recalculated the rate of weight regain based solely on data up to two years after treatment cessation to mirror the duration of follow-up after WMM, leading to slightly higher estimates for the rate of weight regain than in the original paper.^12^ The comparison between WMM and BWMP in this review is indirect. While the population for each review was similar, it is possible that the population included in each review differed slightly (such as degree of obesity and number with comorbidities), and these differences might underpin some of the observed differences in the rate of weight regain. However, evidence from a recent randomised controlled trial that directly compared liraglutide with a BWMP supports our conclusion of faster weight regain after WMM compared with BWMPs.^37^ Finally, we found that few studies were at low risk of bias.

### Interpretation of findings

Weight loss improves cardiovascular risk factors—a recent trial showed that continuous use of semaglutide over four years reduced major cardiovascular disease events in individuals with existing cardiovascular disease.^9^ The benefits of weight loss on diabetes and other cardiovascular disease risk markers were, however, attenuated by weight regain.^10^ Based on projections of observed trends, we estimated that people who stop taking WMMs will regain weight at a rate of 0.4 kg/month, returning to baseline weight by 1.7 years and to no difference from control after 1.4 years. Data were insufficient to analyse changes in cardiometabolic markers after treatment with newer and more effective incretin mimetics, but we have shown that weight regain is faster (0.8 kg/month) and a return to baseline weight projected at 1.5 years after cessation of treatment, implying that the benefits on cardiovascular health will probably also attenuate more rapidly. In England, the National Institute for Health and Care Excellence (NICE) assumed that after cessation of semaglutide or tirzepatide, weight will be regained by two years with current cost effectiveness models for semaglutide based on a return to baseline by three years, where our data suggest weight regain occurs more rapidly. Estimates for rates of weight regain in routine practice are also needed to inform accurate assessments of the cost effectiveness of these treatments.

As obesity is a chronic and relapsing condition, prolonged treatment with WMM may be required to sustain the health benefits. One trial did show successful weight loss maintenance over four years with continuous semaglutide treatment.^9^ In the USA and Denmark, early evidence shows that discontinuation rates outside of clinical trials are around 50% at one year.^14^ ^38^ This evidence suggests that despite their success in achieving initial weight loss, these drugs alone may not be sufficient for long term weight control. Further research is needed to study how to support people to use these drugs effectively, either through prolonged adherence or, possibly, through intermittent periods of treatment. Moreover, the data presented here are averages and, as with BWMPs, a small proportion of participants achieved sustained weight loss. Further research is needed to identify predictors or early indicators of long term success.

Beyond cardiometabolic health, there have been suggestions that WMM may be effective for the treatment of osteoarthritis^39^ ^40^ and the prevention of cognitive decline,^41^ among other conditions. Data were insufficient to analyse these possible benefits of weight loss, but it seems unlikely that improvements in physical functioning or cognitive performance can be sustained if weight is regained. However, WMMs may be beneficial to achieve short term weight loss, perhaps to allow more effective or safer treatment for other conditions (eg, pre-surgery).

### Policy implications

The efficacy of the recent newer incretin mimetic treatments is likely to increase the prescription and use of WMMs, and it is important that individuals are aware of the risk of weight regain after stopping treatment. A survey of US adults found that 45% were interested in the use of GLP-1 receptor agonists for weight management, but this proportion declined to 14% when individuals were informed about weight regain after discontinuing treatment.^42^ In England, the recent NICE quality standard recommends the provision of support after cessation of WMM,^43^ but we found no evidence that such support leads to slower weight regain or that higher levels of behavioural support offered during active weight loss treatment reduced rates of weight regain. Further research is required to optimise support after cessation of WMMs to limit weight regain.

We have previously quantified the rate of weight regain after BWMP.^12^ This analysis allowed us to compare weight regain after the cessation of WMM and BWMP. On average in trials, people lost more weight when using WMMs compared with BWMPs. However, the rate of weight regain was faster after WMMs than after BWMPs and greater initial weight loss was typically associated with faster weight regain.^11^ Nevertheless, in our sensitivity analysis (fig 4), we still observed faster weight regain after WMM for a fixed amount of initial weight loss. Furthermore, the BWMP review found benefits for cardiometabolic health lasting for about five years after the end of the programme. Yet, we found no evidence that this is the case with WMM, as cardiometabolic health markers returned to baseline within 1.4 years after stopping treatment. It is possible that following a BWMP provides people with practical coping skills that they can continue to implement past the end of the intervention to help with weight loss maintenance. On the other hand, although WMM is effective in inducing weight loss, it does so by making behaviour change easier (reduced hunger and increased satiety), which may undermine the value of conscious dietary and physical activity efforts, which are the only recourse after the cessation of WMM. It is important that the use of WMM is considered alongside other treatment options to identify the most appropriate intervention for each individual.^44^

### Conclusions

WMMs are associated with a reduction in weight and improvements in cardiometabolic health that are attenuated soon after treatment ends, with no evidence of benefit 1.7 years after the cessation of treatment. This evidence cautions against short term use of WMMs, emphasises the need for further research into cost effective strategies for long term weight control, and reinforces the importance of primary prevention.

**What is already known on this topic**

The development of highly effective weight management medications (WMMs) has transformed the treatment of obesity
Real world observations estimate that around 50% of people with obesity discontinue WMMs within 12 months
A previous systematic review quantified and compared the rate of weight regain with behavioural weight management programmes (BWMPs)

**What this study adds**

People on average regain weight at a rate of 0.4 kg/month after cessation of WMMs, leading to a projected return to baseline weight after 1.7 years
Although weight loss resulted in improvements in HbA_1c_, fasting glucose, total cholesterol, triglycerides, systolic and diastolic blood pressure, all markers returned to baseline within 1.4 years of treatment cessation
The rate of weight regain after the cessation of WMMs was faster than after the cessation of BWMPs, independent of the amount of weight lost during treatment

## Data Availability

Data may be made available to investigators upon request by email to the corresponding author.

## Web extra

Extra material supplied by authors

## Notes

Contributors: SAJ and DAK are joint senior authors. SW, PA, SAJ, and DAK conceived the study. SW, JS, LW, HK, SH, DW, SM, LH, and DAK contributed to screening and data extraction. SW and DAK had full access to the data. SW, JLO and DAK conducted the data analysis. SW drafted the manuscript. All authors revised the manuscript. SW is the guarantor. The corresponding author attests that all listed authors meet authorship criteria and that no others meeting the criteria have been omitted.

Transparency: The lead author (SW) affirms that the manuscript is an honest, accurate, and transparent account of the study being reported; that no important aspects of the study have been omitted; and that any discrepancies from the study as planned (and, if relevant, registered) have been explained; the protocol was registered in advance and all changes were detailed to the protocol record with reasons given.

Dissemination to participants and related patient and public communities: The results of this study have been shared with members of the Health Behaviours Weight Management Panel. People with experience of weight loss drugs have been invited to help in the development of our dissemination plan.

Provenance and peer review: Not commissioned; externally peer reviewed.
