# Supplementary material for: Weight regain after cessation of medication for weight management: systematic review and meta-analysis
Source: BMJ. 2026 Jan 7;392:e085304. doi: 10.1136/bmj-2025-085304 (PMC12776922; doi:10.1136/bmj-2025-085304)

Weight regain following the cessation of medication for weight management: a systematic review and meta-analysis.

Supplementary Material

Table of Contents

Supplementary Table 1. Medline search strategy. 3

Supplementary Table 2. Details of the intervention, comparator and follow-up support of the studies included in the analysis. 5

Supplementary Table 3. Risk of Bias 14

Supplementary Table 4. Results of the primary and sensitivity analysis 16

Supplementary Table 5. Comparison of the PICO for the present review and the review of BWMPs. 17

Supplementary Table 6. GRADE assessment. 19

Supplementary Figure 1. Evidence search and selection. 20

Supplementary Figure 2. Meta-regression of all randomised controlled trials. 21

Supplementary Figure 3. Sensitivity analysis assessing whether weight of regain differed when patients were offered low (nothing, leaflet, self-help CBT, dietary and physical activity advice at baseline) or high (individual or group counselling throughout treatment, structured behaviour change programme) level support during treatment with WMM (A) or incretin-based therapies (B) analysed with a mixed-model (Model 1). 22

Supplementary Figure 4. Sensitivity analysis assessing whether the rate of weight regain differed when patients where offered behavioural support or no support after WMM 23

Supplementary Figure 5. Sensitivity analysis assessing whether the rate of weight regain differed when patients where offered active treatment (behavioural support/metformin) or non-active treatment (nothing/placebo) after WMM 24

Supplementary Figure 6. Funnel plot for randomised controlled trials. 25

Supplementary Figure 7. Funnel plot for single arm trials. 26

Supplementary Figure 8. Comparison of linear (blue) and curvilinear (red) models to assess the rate of weight regain after cessation of WMM 27

Supplementary Figure 9. Comparison of linear (blue) and curvilinear (red) models to assess the rate of weight regain after cessation of incretin mimetics 28

Supplementary Figure 10. Comparison of linear (blue) and curvilinear (red) models to assess the rate of weight regain after cessation of newer and more effective incretin mimetics. 29

Supplementary Figure 11. Forest plot of all timepoints from studies using WMM included in the mixed model (Figure 1a). Data are plotted as weight change (kg) from baseline. 30

Supplementary Figure 12. Forest plot of all timepoints from studies using incretin mimetic therapies included in the mixed model (Figure 1b). Data are plotted as weight change (kg) from baseline 33

Supplementary Figure 13. Forest plot of all timepoints from studies using newer and more effective incretin mimetic therapies included in the mixed model (Figure 1c). Data are plotted as weight change (kg) from baseline. 35

Supplementary Figure 14. Forest plot of all timepoints from RCTs using WMM included in the mixed model (Figure 2a). Data are plotted as difference in weight change (kg) from baseline between intervention and control. 36

Supplementary Figure 15. Forest plot of all timepoints from RCTs using incretin mimetic therapies included in the mixed model (Figure 2b). Data are plotted as difference in weight change (kg) from baseline between intervention and control. 39

Supplementary Figure 16. Forest plot of all timepoints from RCTs using newer and more effective incretin mimetic therapies included in the mixed model (Figure 2c). Data are plotted as difference in weight change (kg) from baseline between intervention and control. 41

Supplementary Table 1. Medline search strategy.

1	Overweight/dt [Drug Therapy]
2	obesity/dt or obesity, abdominal/dt or obesity, maternal/dt or obesity, morbid/dt
3	overweight/ or obesity/ or obesity, abdominal/ or obesity, maternal/ or obesity, morbid/
4	(obes* or overweight).ti,kf.
5	3 or 4
6	Anti-Obesity Agents/
7	((((pharmacolog* or drug*) adj3 (therap* or treatment? or agent? or intervention?)) or pharmacotherap*) not (non-pharmacolog* adj3 (therap* or treatment? or agent? or intervention?))).ti,kf.
8	((antiobesity or anti-obesity) adj3 (medication? or medicine? or drug? or agent?)).ti,kf.
9	(liraglutide or saxenda or victoza or semaglutide or wegovy or tirzepatide or exenatide or bydureon or byetta or lixisenatide or albiglutide or tanzeum or eperzan or dulaglutide or trulicity or phentermine or adipex-p or suprenza or topiramate or topamax or qsymia or lorcaserin or belviq or (naltrexone and bupropion) or sibutramine or rimonabant or benzphetamine or diethylpropion or tenuate or phendimetrazine or bontril or orlistat or xenical).ti,ab,kf.
10	6 or 7 or 8 or 9
11	body weight changes/ or weight loss/ or weight gain/
12	Body Mass Index/
13	((bodyweight or weight) adj3 (loss or losing or lose or lost or chang* or reduc* or maintain* or maintenance or manage* or increas* or gain* or regain*)).ti,ab,kf.
14	11 or 12 or 13
15	5 and 10 and 14
16	1 and 14
17	2 and 14
18	15 or 16 or 17
19	exp randomized controlled trial/
20	controlled clinical trial.pt.
21	comparative study/
22	multicenter study.pt.
23	pragmatic clinical trial.pt.
24	(randomis* or randomiz* or randomly).ti,ab.
25	groups.ab.

26	(trial or multicenter or multi center or multicentre or multi centre).ti.
27	(intervention? or effect? or impact? or controlled or control group? or (before adj5 after) or (pre adj5 post) or ((pretest or pre test) and (posttest or post test)) or quasiexperiment* or quasi experiment* or pseudo experiment* or pseudoexperiment* or cohort? or evaluat* or time series or time point? or repeated measur*).ti,ab.
28	non-randomized controlled trials as topic/
29	interrupted time series analysis/
30	controlled before-after studies/
31	or/19-30
32	exp animals/
33	humans/
34	32 not (32 and 33)
35	review.pt.
36	meta analysis.pt.
37	news.pt.
38	comment.pt.
39	editorial.pt.
40	cochrane database of systematic reviews.jn.
41	comment on.cm.
42	(systematic review or literature review).ti.
43	or/34-42
44	31 not 43
45	18 and 44

Supplementary Table 2. Details of the intervention, comparator and follow-up support of the studies included in the analysis.

			Intervention			Comparator			Follow-up	
**Author**	**Date**	**Design**	***n***	**Details**	**Duration (weeks)**	***n***	**Details**	**Duration (weeks)**	**Details**	**Duration (weeks)**
Aronne	2023	RCT*	335	Tirzepatide (15 mg/wk) + patients received lifestyle counseling by a qualified health care professional throughout the study to encourage adherence to a healthy 500 kcal/d deficit diet and at least 150 minutes of physical activity per week.	36	-	-	-	Placebo + Lifestyle support continued from the intervention.	52
Brownell	1981	RCT	69	Fenfluramine (160 mg/d) + All participants were prescribed a treatment manual which emphasised behaviour change to control food intake.	16	43	Behavioural - All participants were prescribed a treatment manual which emphasised behaviour change to control food intake.	16	Behavioural - Lifestyle support continued from the intervention.	52
Craighead[a]	1984	RCT	16	Fenfluramine (160 mg/d) + A standard 1,000-1,200 kcal/day balanced diet Information regarding several varieties of exercise programs was provided.	16	16	Behavioural - A standard 1,000-1,200 kcal/day balanced diet Information regarding several varieties of exercise programs was provided.	16	Nothing	52
Craighead[b]	1984	RCT	14	Fenfluramine (160 mg/d) + Behaviour therapy was given in a highly structured program including self-monitoring, stimulus control, modification of eating behavior, self-reinforcement, cognitive restructuring,	16	16	Same as above.	16	Nothing	52

				contingency contracting, and exercise management						
Craighead[c]	1984	RCT	13	Fenfluramine (160 mg/d) + same as above.	8	16	Same as above.	16	Nothing	60
Craighead[d]	1984	RCT	15	Fenfluramine (160 mg/d) + same as above.	8	16	Same as above.	16	Nothing	52
Craighead[a]	1981	RCT	40	Fenfluramine (120 mg/d) + patients received supportive group counselling designed to reproduce the nonspecific elements of the behaviour therapy program, such as group support, interest and attention	26	40	Behavioural - Behaviour therapy was given in a highly structured program including self-monitoring, stimulus control, modification of eating behavior, self-reinforcement, cognitive restructuring, contingency contracting, and exercise management	26	Behavioural – actual support provided is unclear.	52
Craighead[b]	1981	RCT	34	Fenfluramine (120 mg/d) + Behaviour therapy given in a highly structured program including self-monitoring, stimulus control, modification of eating behavior, self-reinforcement, cognitive restructuring, contingency contracting, and exercise management	26	40	Same as above.	26	Behavioural – actual support provided is unclear.	52
Croghan[a]	2016	RCT	14	Lorcaserin (20 mg/d)	12	15	LLT	12	Nothing	12
Croghan[b]	2016	RCT	15	Lorcaserin (20 mg/d)	12	15	LLT	12	Nothing	12
Davidson	1999	RCT	657	Orlistat (360 mg/d) + patients were prescribed a 500-800 kcal/d deficit with 30% energy coming from fat.	52	223	Placebo + patients were prescribed a 500-800 kcal/d deficit with 30% energy coming from fat.	52	Placebo + patients were prescribed a weight maintenance diet and encouraged to walk 3-5 times per week.	52
Davies[a]	2015	RCT	423	Liraglutide (3.0 mg/d) + patients were advised to	56	212	Placebo + patients were advised to follow a 500 kcal/day deficit	56	Nothing	12

				follow a 500 kcal/day deficit and increase physical activity to at least 150 min per week and provided with monthly counselling on diet and exercise.			and increase physical activity to at least 150 min per week and provided with monthly counselling on diet and exercise.			
Davies[b]	2015	RCT	211	Liraglutide (1.8 mg/d) + same as above	56	212	Same as above	56	Nothing	12
Dawson	2011	RCT	8	Rimonabant (20 mg/d)	52	8	Placebo	52	Metformin	12
Early	2007	RCT*	55	Sibutramine (15 mg/d) + patients were prescribed a LCD (1200–1500 kcal/d) and encouraged to exercise.	12	-	-	-	Behavioural - LCD (1500 total kcal/d consisting of three low-calorie meals).	36
Ferjan[a]	2017	RCT*	12	Liraglutide (3.0 mg/d)	12	-	-	-	Metformin	12
Ferjan[b]	2017	RCT*	12	Liraglutide (3.0 mg/d)	12	-	-	-	Metformin + sitagliptin	12
Grilo[a]	2014	RCT	26	Sibutramine (15 mg/d)	16	27	Placebo	16	Nothing	52
Grilo[b]	2014	RCT	26	Sibutramine (15mg/d) + self-help CBT.	16	27	Placebo	16	Nothing	52
Jastreboff[a]	2024	RCT	247	Tirzepatide (5.0 mg/wk) + patients received regular counselling focusing on a 500 kcal/day deficit and at least 150 min/wk of physical activity.	176	270	Placebo + patients received regular counselling focusing on a 500 kcal/day deficit and at least 150 min/wk of physical activity.	72	Nothing	17
Jastreboff[b]	2024	RCT	262	Tirzepatide (10 mg/wk) + same as above.	176	270	Same as above.	72	Nothing	17
Jastreboff[c]	2024	RCT	253	Tirzepatide (15 mg/wk) + same as above.	176	270	Same as above.	72	Nothing	17
Jensterle	2024	OBS	25	Semaglutide (1 mg/wk) + metformin + promotion of healthy lifestyle intervention.	16	-	-	-	Metformin + promotion of healthy lifestyle intervention.	104

Karhunen	2000	RCT	36	Orlistat (360 mg/d) + patients received regular counselling on a hypocaloric diet with 30% energy coming from fat (600 kcal/d deficit). Calories were reduced by a further 300 kcal/day at week 24.	52	36	Placebo + patients received regular counselling on a hypocaloric diet with 30% energy coming from fat (600 kcal/d deficit). Calories were reduced by a further 300 kcal/day at week 24.	52	Placebo + patients were prescribed a weight maintenance diet.	52
Khoo	2019	RCT	15	Liraglutide (3.0 mg/d)	26	15	Behavioural – patients were prescribed a 400 kcal/d deficit supported by dieticians, physical trainers and physicians.	26	Behavioural – patients were provided with resources on portion control and energy restriction and advised to increase physical activity to 150-200 mins per week.	26
Kwon[a]	2022	RCT*	24	Orlistat + phentermine (360 mg/d + 37.5 mg/d) + patients were advised to follow a 500 kcal/d deficit and engage in light to moderate exercise 3-5 times per week.	12	-	-	-	Nothing	24
Kwon[b]	2022	RCT*	27	Phentermine (37.5 mg/d) + same as above.	12	-	-	-	Nothing	24
Lau[a]	2021	RCT	101	Cagrilintide (0.3 mg/wk) + All participants received dietary and physical activity counselling, aiming to achieve a 500 kcal/d deficit and 150 min of physical activity per week.	26	101	Placebo + All participants received dietary and physical activity counselling, aiming to achieve a 500 kcal/d deficit and 150 min of physical activity per week.	26	Nothing	6
Lau[b]	2021	RCT	100	Cagrilintide (0.6 mg/wk) + same as above.	26	101	Same as above.	26	Nothing	6
Lau[c]	2021	RCT	102	Cagrilintide (1.2 mg/wk) + same as above.	26	101	Same as above.	26	Nothing	6

Lau[d]	2021	RCT	102	Cagrilintide (2.4 mg/wk) + same as above.	26	101	Same as above.	26	Nothing	6
Lau[e]	2021	RCT	101	Cagrilintide (4.5 mg/wk) + same as above.	26	101	Same as above.	26	Nothing	6
Lau[f]	2021	RCT	99	Liraglutide (3.0 mg/d) + same as above.	26	101	Same as above.	26	Nothing	6
leRoux	2017	RCT	1505	Liraglutide (3.0 mg/d) + participants received dietary and physical activity counselling, aiming to achieve a 500 kcal/d deficit and increase physical activity to at least 150 min per week.	160	749	Placebo + participants received dietary and physical activity counselling, aiming to achieve a 500 kcal/d deficit and increase physical activity to at least 150 min per week.	160	Behavioural – Lifestyle support continued from the intervention.	12
Liang	2014	SAT	10	Topiramate (50 mg/d) + patients on the ward were provided food (~2000 kcal/d) and given 30 min of daily moderate intensity exercise.	16	-	-	-	Nothing	52
Marbury[a]	1996	RCT	85	Dexfenfluramine (10 mg/d) + Patients were placed on calorically restricted diets based on sex and initial weight, and diet counselling was included at each study visit from baseline to the end of the follow-up period.	12	85	Behavioural - Patients were placed on calorically restricted diets based on sex and initial weight, and diet counselling was included at each study visit from baseline to the end of the follow-up period.	12	Behavioural - Lifestyle support continued from the intervention.	4
Marbury[b]	1996	RCT	82	Dexfenfluramine (30 mg/d) + same as above.	12	85	Same as above.	12	Behavioural – same as above.	4
Marbury[c]	1996	RCT	87	Dexfenfluramine (60 mg/d) + same as above.	12	85	Same as above.	12	Behavioural – same as above.	4
McGowan	2024	RCT	138	Semaglutide (2.4 mg/wk) + patients were offered individual diet and physical activity counselling by a	52	69	Placebo + patients were offered individual diet and physical activity counselling by a	52	Behavioural - patients were offered healthy lifestyle counselling	28

				dietitian or a similar qualified health-care professional.			dietitian or a similar qualified health-care professional.		according to standard clinical practice and country-specific guidelines	
Moolla	2025	RCT	15	Liraglutide (1.8 mg/d)	12	14	Behavioural – patients followed a 500 kcal/day deficit, decreased consumption of fat (to <30% of dietary calorie intake) alongside increased fibre intake, principally through increased consumption of vegetables and fruit.	12	Nothing	12
Napolitano	2012	RCT	19	Sibutramine (10 mg/d) + patients were prescribed a 600 kcal/d deficit by a dietitian and received dietetic consultation at each visit.	12	20	Placebo + patients were prescribed a 600 kcal/d deficit by a dietitian and received dietetic consultation at each visit.	12	Nothing	12
Oneil[a]	2018	RCT	103	Semaglutide (0.05 mg/wk) + participants received dietary and physical activity counselling, aiming to achieve a 500 kcal/d deficit and 150 min of physical activity per week.	52	136	Placebo + participants received dietary and physical activity counselling, aiming to achieve a 500 kcal/d deficit and 150 min of physical activity per week.	52	Nothing	7
Oneil[b]	2018	RCT	102	Semaglutide (0.4 mg/wk) + same as above.	52	136	Same as above.	52	Nothing	7
Oneil[c]	2018	RCT	102	Semaglutide (0.3 mg/wk FE) + same as above.	52	136	Same as above.	52	Nothing	7
Oneil[d]	2018	RCT	103	Semaglutide (0.4 mg/wk FE) + same as above.	52	136	Same as above.	52	Nothing	7
Oneil[e]	2018	RCT	103	Liraglutide (3.0 mg/d) + same as above.	52	136	Same as above.	52	Nothing	7

Pi-Sunyer	2006	RCT	1219	Rimonabant (20 mg/d) + patients were prescribed a 600 kcal/d deficit and encouraged to increase their physical activity.	52	607	Placebo + patients were prescribed a 600 kcal/d deficit and encourage to increase their physical activity.	52	Placebo + patients were prescribed a 600 kcal per day deficit and encourage to increase their physical activity.	52
Pi-Sunyer	2015	RCT	959	Liraglutide (3.0 mg/d) + patients received counselling aiming to achieve a 500 kcal/d deficit and 150 min of physical activity per week.	56	223	Placebo + patients received counselling aiming to achieve a 500 kcal/d deficit and 150 min of physical activity per week.	56	Behavioural - Lifestyle support continued from the intervention.	12
Rodin	1988	RCT	16	Diethylpropion hydrochloride (75 mg/d) + weekly CBT focusing on eating behaviour, physical activity, self-cognition and relapse training	11	16	Placebo + weekly CBT focusing on eating behaviour, physical activity, self-cognition and relapse training	11	Nothing	32
Rosenstock[a]	2023	RCT	98	Tirzepatide (5.0 mg/wk) + patients were given general counselling on diet and exercise.	40	57	Placebo + patients were given general counselling on diet and exercise.	40	Nothing	4
Rosenstock[b]	2023	RCT	90	Tirzepatide (10 mg/wk) + same as above.	40	57	Same as above.	40	Nothing	4
Rosenstock[c]	2023	RCT	78	Tirzepatide (15 mg/wk) + same as above.	40	57	Same as above.	40	Nothing	4
Rubino	2021	RCT*	803	Semaglutide (2.4 mg/wk) + patients received monthly counselling aiming to achieve a 500 kcal/d deficit and 150 minutes of physical activity a week.	20	-	-	-	Behavioural - Lifestyle support continued from the intervention.	48
Samp[a]	2015	RCT*	18	Orlistat (360 mg/d) + patients were educated on the calorie content of different foods and provided with advice to increase physical activity.	36	-	-	-	Nothing	12

				Patients had regular diet and physical activity consultations.						
Samp[b]	2015	RCT*	18	Sibutramine (15 mg/d) + same as above.	36	-	-	-	Nothing	12
Sathyapalan	2009	RCT	10	Rimonabant (20 mg/d)	12	10	Metformin	12	Metformin	12
Sjostrom	1998	RCT	138	Orlistat (360 mg/d) + patients were prescribed a hypocaloric diet with 30% energy coming from fat (600 kcal/d deficit). Calories were reduced by a further 300 kcal/d at week 24.	52	123	Placebo + patients were prescribed a hypocaloric diet with 30% energy coming from fat (600 kcal/d deficit). Calories were reduced by a further 300 kcal/d at week 24.	52	Placebo + patients were prescribed a weight maintenance eucaloric diet.	52
Smith	2010	RCT	283	Lorcaserin (20 mg/d) + patients received regular counselling, aiming to achieve a 600 kcal/d deficit and daily moderate exercise for 30 min.	52	1499	Placebo + patients received regular counselling, aiming to achieve a 600 kcal/d deficit and daily moderate exercise for 30 min.	52	Behavioural - Lifestyle support continued from the intervention.	52
Svensson	2019	RCT	47	Liraglutide (1.8 mg/d)	16	51	Placebo	16	Nothing	52
Wadden	2013	RCT	212	Liraglutide (3 mg/d) + participants were prescribed a 500 kcal/d deficit. Face-to-face counselling visits were provided weekly for the first month and then monthly until the end of the intervention.	56	210	Placebo + participants were prescribed a 500 kcal/d deficit. Face-to-face counselling visits were provided weekly for the first month and then monthly until the end of the intervention.	56	Nothing	12
Wilding	2022	RCT	228	Semaglutide (2.4 mg/wk) + Counselling every 4 weeks on diet (500 kcal/d deficit) and physical activity (150 minutes per week)	68	99	Placebo + Counselling every 4 weeks on diet (500 kcal/d deficit) and physical activity (150 minutes per week)	68	Nothing	52

Woo[a]	2007	RCT*	28	Orlistat (360 mg/d) + patients were asked to continue their normal diet.	26	-	-	-	Nothing	26
Woo[b]	2007	RCT*	27	Orlistat (360 mg/d) + same as above.	26	-	-	-	Behavioural – patients were provided with peer group support sessions that focused on dietary management, physical activity and exercise management.	26

CBT = cognitive behavioural therapy. FE = fast escalation. LCD = low calorie diet. LLT = Low-level laser therapy. * these studies were RCTs by design but did not have a placebo group during both the treatment *and* off-treatment follow up phase and were therefore not included in the RCT analysis.

Supplementary Table 3. Risk of Bias assessed using the Cochrane Risk of Bias 2 tool (RCTs) and the ROBINS-I toll for all other trials.

Author	Date	D1	D2	D3	D4	D5	Overall
Aronne	2023	Low	Low	Low	Low	Low	Low
Brownell	1981	High	Some concerns	Low	High	Some concerns	High
Craighead	1984	High	Some concerns	Low	Some concerns	Some concerns	High
Craighead	1981	High	Some concerns	Low	Some concerns	Some concerns	High
Croghan	2016	Some concerns	Some concerns	Low	Low	Some concerns	High
Davidson	1999	Some concerns	Low	Some concerns	Low	Some concerns	High
Davies	2015	Low	Low	Low	Low	Low	Low
Dawson	2011	Some concerns	High	Low	Low	Low	High
Early	2007	High	Some concerns	Low	Low	Some concerns	High
Ferjan	2017	High	Low	Low	Low	Low	High
Grilo	2014	Low	Some concerns	Some concerns	Some concerns	Some concerns	High
Jastreboff	2024	Some concerns	Low	Some concerns	Low	Low	Some concerns
Karhunen	2000	Some concerns	Low	Low	Low	Low	Some concerns
Khoo	2019	Some concerns	Some concerns	Low	Low	Some concerns	High
Kwon	2022	Low	Low	Some concerns	Low	Low	Some concerns
Lau	2021	Low	Low	Low	Low	Low	Low
leRoux	2017	Low	Low	Low	Low	Low	Low
Marbury	1996	Low	Some concerns	Low	Low	Some concerns	High
McGowan	2024	Low	Low	Low	Low	Low	Low
Moolla	2025	Low	Low	Low	Low	Low	Low
Napolitano	2012	High	Some concerns	Low	Low	Some concerns	High
O'Neil	2018	Low	Low	Low	Low	Low	Low
Pi-Sunyer	2006	High	High	High	High	High	High
Pi-Sunyer	2015	Low	Low	Some concerns	Low	Low	Some concerns
Rodin	1988	Some concerns	Low	Low	Low	Some concerns	High
Rosenstock	2023	Low	Low	Some concerns	Low	Some concerns	Some concerns
Rubino	2021	Low	Low	Low	Low	Low	Low
Samp	2015	Some concerns	High	High	Low	High	High
Sathyapalan	2009	Some concerns	High	Low	Some concerns	Some concerns	High
Sjostrom	1998	Low	Low	Low	Low	Low	Low
Smith	2010	High	Low	High	Low	Low	High
Svensson	2019	Low	Low	Low	Low	Low	Low
Wadden	2013	Low	Low	Low	Low	Low	Low
Wilding	2022	Low	Low	Low	Low	Low	Low
Woo	2007	Some concerns	Some concerns	Low	Some concerns	Some concerns	High

Author	Date	D1	D2	D3	D4	D5	D6	D7	Overall
Jensterle	2024	Critical	Critical		Critical	Critical	Low	Moderate	Critical
Liang	2014	Low	Low		Low	Low	Moderate	Moderate	Serious

Supplementary Table 4. Results of the primary and sensitivity analysis including studies with a low risk of bias.

Studies analysed	Primary analysis		Sensitivity analysis	
	**Weight loss (kg)**	**Rate of weight regain (kg/month)**	**Weight loss (kg)**	**Rate of weight regain (kg/month)**
All Studies				
All medication[1]	8.3 (7.2-9.5)	0.39 (0.34-0.48)	10.2 (8.4-12.0)	0.65 (0.52-0.74)
All incretin mimetics[1]	10.1 (8.2-11.9)	0.52 (0.39-0.65)	9.7 (7.0-12.5)	0.69 (0.56-0.82)
New incretin mimetics[1]	14.7 (11.1-18.4)	0.82 (0.69-0.94)	15.7 (12.6-18.9)	0.82 (0.69-0.91)
RCTs				
All medication[1]	5.7 (4.4-6.9)	0.34 (0.26-0.39)	6.8 (5.0-8.7)	0.39 (0.30-0.52)
All medication[2]	6.2 (4.7-7.8)	0.39 (0.21-0.65)	5.9 (3.8-8.1)	0.17 (-0·18-0.61)
All incretin mimetics[1]	8.0 (6.1-9.9)	0.61 (0.43-0.78)	6.2 (3.7-8.7)	0.56 (0.39-0.74)
New incretin mimetics[1]	12.3 (8.6-15.9)	0.78 (0.56-0.99)		

[1] indicates analysis by mixed effects model, [2] indicates analysis by meta-regression.

Supplementary Table 5. Comparison of the PICO for the present review and the review of BWMPs.

	WMM	BWMP
Population	Adults (≥18 years) with overweight and/or obesity at study start. We will follow the definitions for overweight/obesity outlined in each individual study.<br>Excluding pregnancy.	Adults (≥18 years) with overweight or obesity at study start (BMI of ≥25 kg/m2 or a BMI of ≥23 kg/m2 in Asian populations).<br>Excluding children and women who are pregnant.
Intervention	Follow-up (≥ 4 weeks) after cessation of pharmacological weight loss intervention lasting ≥ 8 weeks.<br>• Including studies that have combined pharmacological and behavioural interventions (ranging from leaflets to total dietary replacement).<br>• Including studies using medication currently or previously licenced for weight loss or where there is reason to believe that the medication studied shares a class effect with a currently or previously licensed medication. These include orlistat, GLP-1s receptor agonists (liraglutide, semaglutide, tirzepatide, exenatide, lixisenatide, albiglutide, dulaglutide), phentermine and topiramate, lorcaserin, naltrexone and bupropion, sibutramine, rimonabant, phentermine, benzphetamine, diethylpropion, phendimetrazine, fenfluramine and dexfenfluramine.	Behavioural weight management programmes (BWMP), defined as any weight management programme aiming to achieve weight loss through changes to diet and/or activity. This will include, but is not limited to:<br>• Multi or single component behavioural counselling programmes: programmes which aim to achieve changes in a participant's diet (single component), exercise (single component), or both (multicomponent) through person-to-person contact.<br>• Self-help programmes: programmes used by individuals for a weight loss attempt not assisted by health care professionals, counsellors, or any other kind of person to person support; this includes, but is not limited to, automated internet interventions, mobile phone applications, and printed material.<br>• Partial or total diet replacement programmes: programmes providing replacements for some (partial) or all (total) meals or snacks consumed during the day and designed to achieve an energy restricted diet (may typically include commercial products used for the purpose of replacing specific meals, or pre-packaged or portion-controlled foods intended to replace usual meals or snacks).<br><br>Interventions which are not explicitly identified as 'weight loss' will be included as long as they fall into the above types (e.g. by their nature entail a calorie deficit). We will exclude studies of weight loss medications, surgery, acupuncture, and nutrient supplementation.

Comparator	Not applicable for single-arm trials and comparative trials of medication for weight loss (as these will be treated as single-arm trials). For controlled trials, we will examine regain relative to any comparator but group these by nature of the comparator (e.g., non-pharmacological weight loss intervention, placebo). We anticipate that any co-interventions will be common across arms, but, if not, will exclude any active comparator that is not present in the group treated with weight loss medication from the main analysis.	A comparator group of lesser intensity or another BWMP, as long as the behavioural interventions differed on a variable of interest in our analysis (e.g. programme type, length, duration, frequency, mode of delivery). Comparators that involve weight loss medications and/or surgery will be excluded.
Outcomes	Rate of weight regain after WMM cessation (kg).	Rate of weight change after programme end (kg).
Study	Randomised trials, non-randomised comparative trials, single-arm trials, and prospective and retrospective observational cohorts. WMM treatment had to last ≥ 8 weeks with a follow up of ≥ 4 weeks after the cessation of the medication.	Randomized controlled trials only. To be included, studies must follow-up participants for ≥ 12 months from baseline and include a measure of weight change at programme end and after programme end.

Supplementary Table 6. GRADE assessment**.**

Outcomes	Studies	N (randomised)	Risk of bias	Inconsistency	Indirectness	Imprecision	Publication bias	Certainty of evidence (GRADE)	Comments
Rate of weight regain	37 (35 RCTs)	9,199	Serious	Not serious	Not serious	Not serious	Not serious	⨁⨁⨁◯ Moderate ¶	Patients who stop taking WMM are estimated to regain weight at a rate of 0.4 kg/month. This estimate of weight regain is faster after incretin mimetics (0.5 kg/month) and newer and more effective incretin mimetics (0.8kg/month).

GRADE Working Group grades of evidence
High certainty: we are very confident that the true effect lies close to that of the estimate of the effect.
Moderate certainty: we are moderately confident in the effect estimate: the true effect is likely to be close to the estimate of the effect, but there is a possibility that it is substantially different.is
Low certainty: our confidence in the effect estimate is limited: the true effect may be substantially different from the estimate of the effect.
Very low certainty: we have very little confidence in the effect estimate: the true effect is likely to be substantially different from the estimate of effect.

¥ Most studies were judged to have a high risk of bias or some concerns. This main reasons for concern were lack of clarity in the randomisation process or lack of prespecified analytical/reporting plan. It was judged that these biases were unlikely to significantly lower the confidence in our evidence, so we opted to downgrade by one in the domain of risk of bias.
¶ While the direction of effect is the same across all studies, the point estimates varied across studies with large heterogeneity. This heterogeneity is largely explained by the differences in the degree of weight loss induced which can be attributed to variations in the medication used and the length of the intervention (73% of total variation explained). Therefore, we opted not to downgrade in the domain of inconsistency.

Supplementary Figure 1. Evidence search and selection.

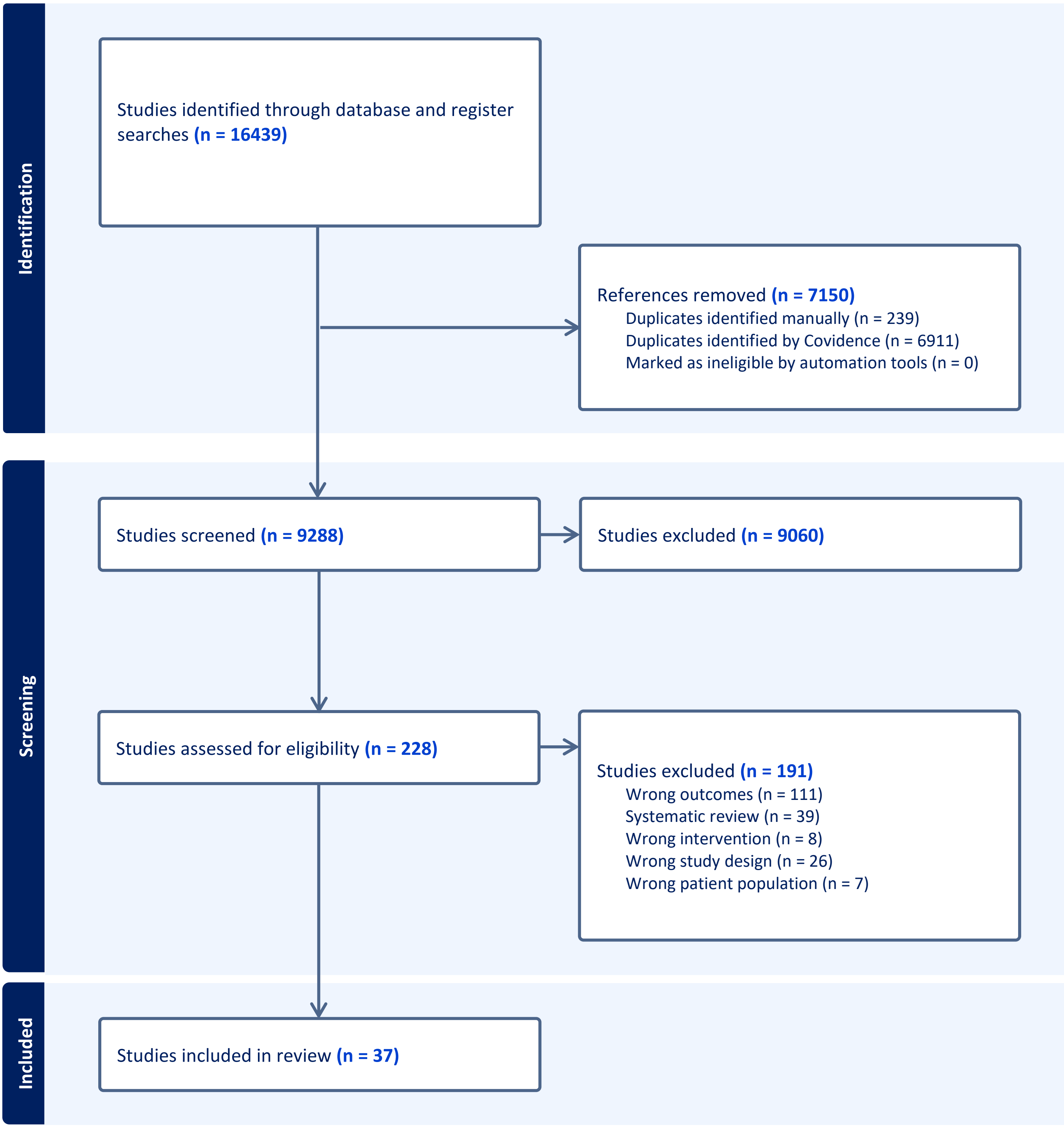

Supplementary Figure 2. Meta-regression of all randomised controlled trials.

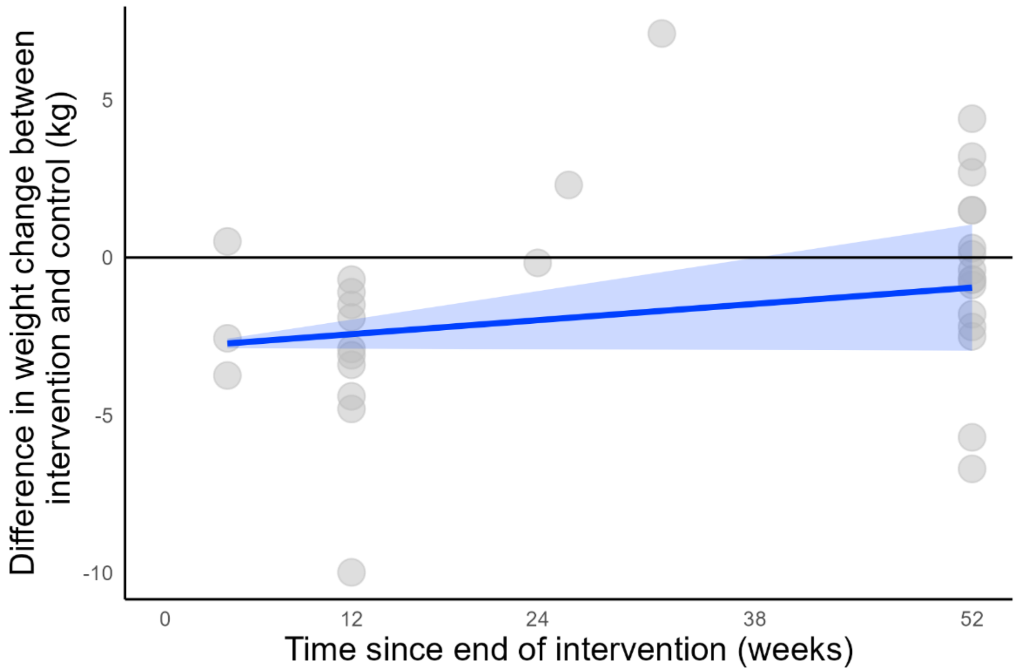

Supplementary Figure 3. Sensitivity analysis assessing whether weight of regain differed when patients were offered low (nothing, leaflet, self-help CBT, dietary and physical activity advice at baseline) or high (individual or group counselling throughout treatment, structured behaviour change programme) level support during treatment with WMM (A) or incretin-based therapies (B) analysed with a mixed-model (Model 1).

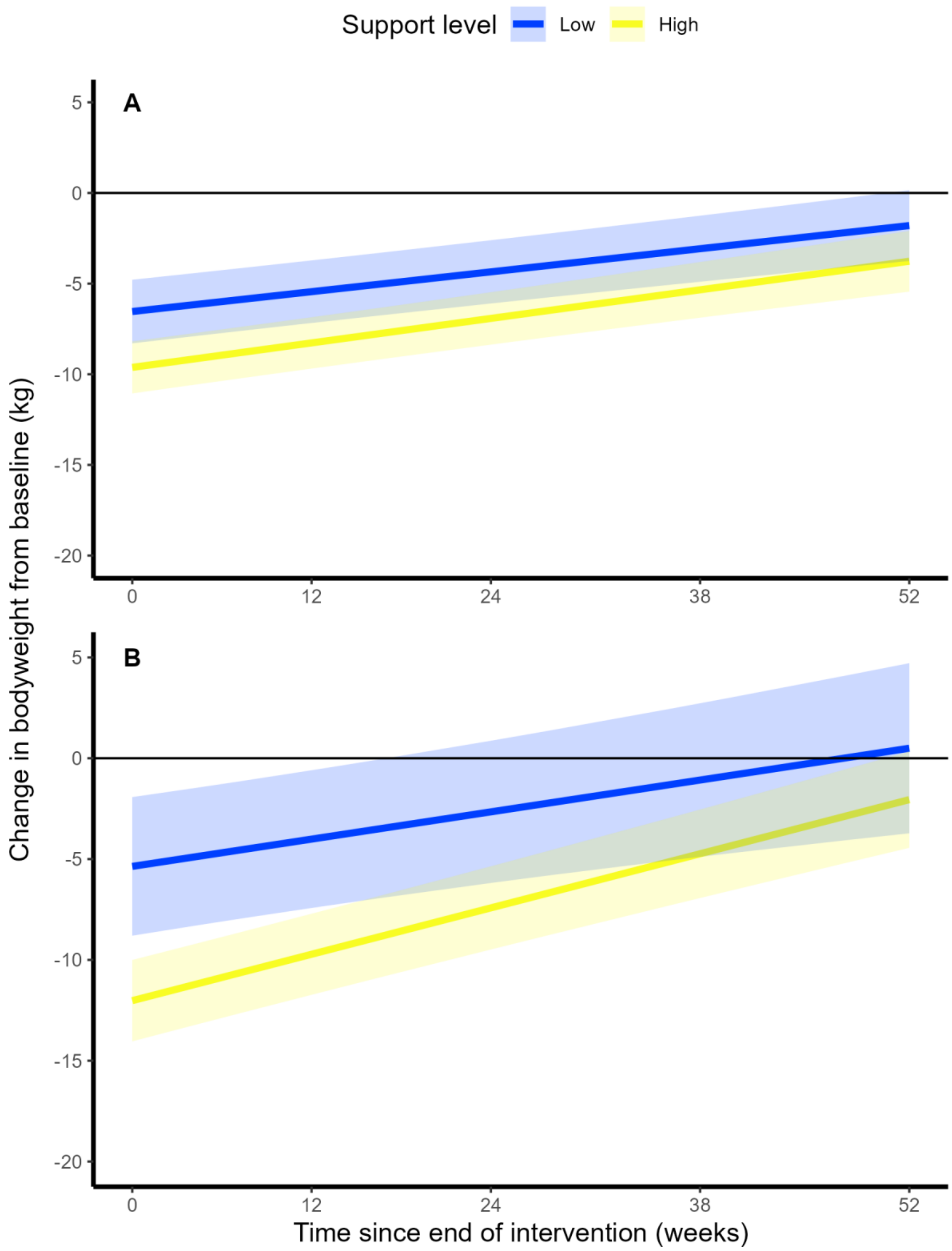

Supplementary Figure 4. Sensitivity analysis assessing whether the rate of weight regain differed when patients where offered behavioural support or no support after WMM analysed with a mixed-model (Model 1).

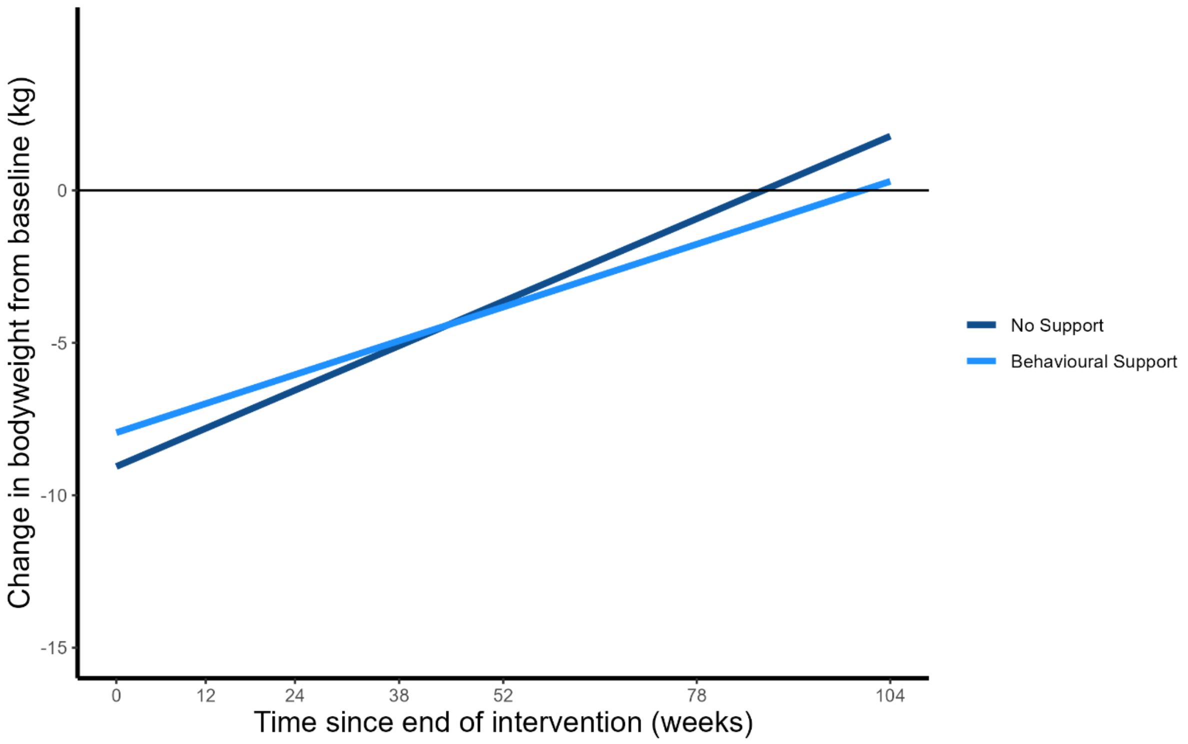

Supplementary Figure 5. Sensitivity analysis assessing whether the rate of weight regain differed when patients where offered active treatment (behavioural support/metformin) or non-active treatment (nothing/placebo) after WMM analysed with a mixed-model (Model 1).

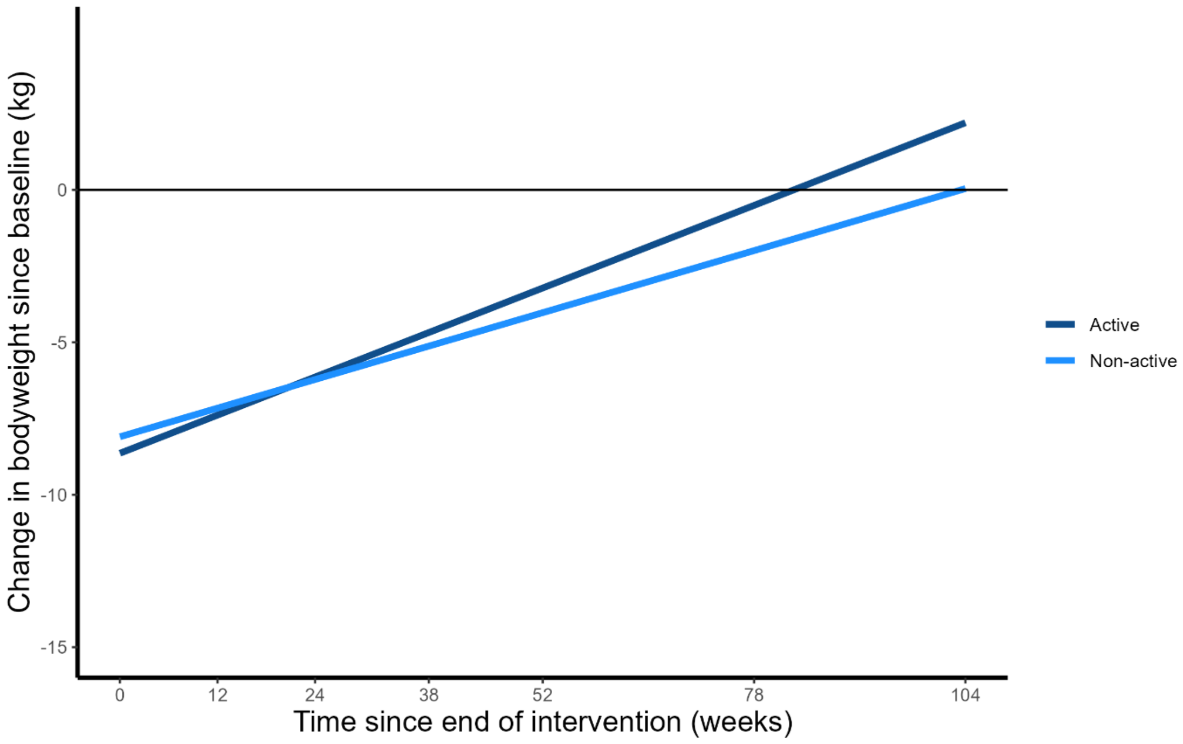

Supplementary Figure 6. Funnel plot for randomised controlled trials.

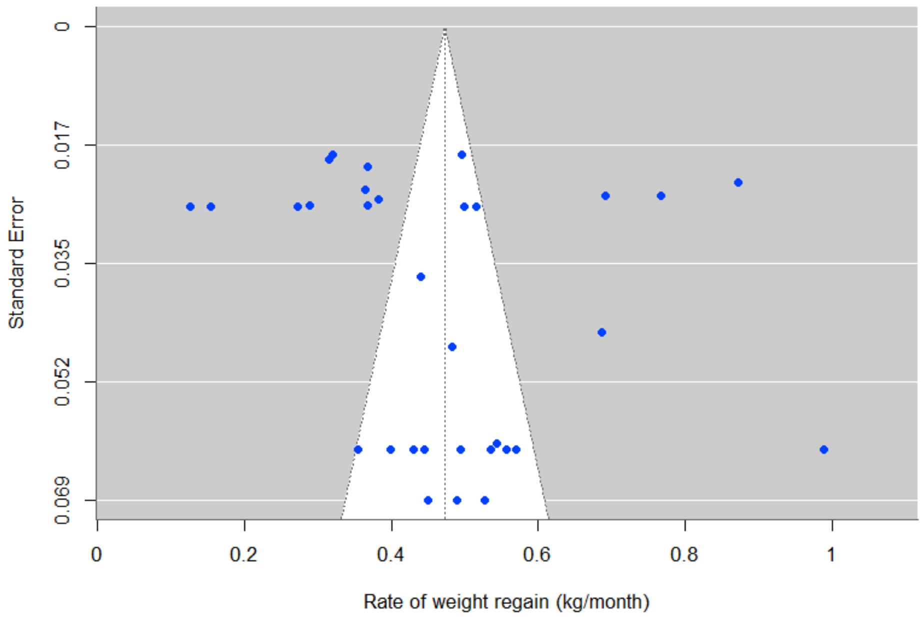

Supplementary Figure 7. Funnel plot for single arm trials.

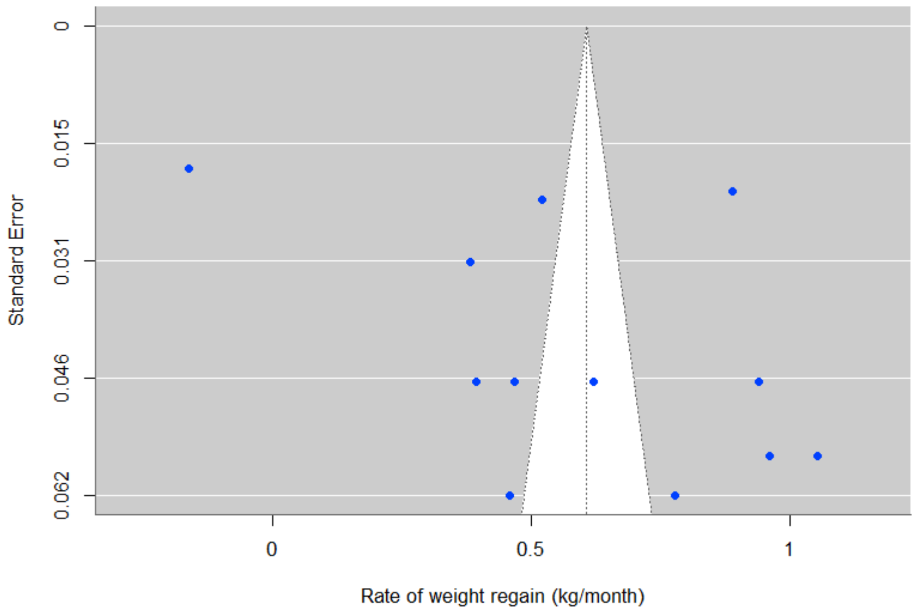

Supplementary Figure 8. Comparison of linear (blue) and curvilinear (red) models to assess the rate of weight regain after cessation of WMM.

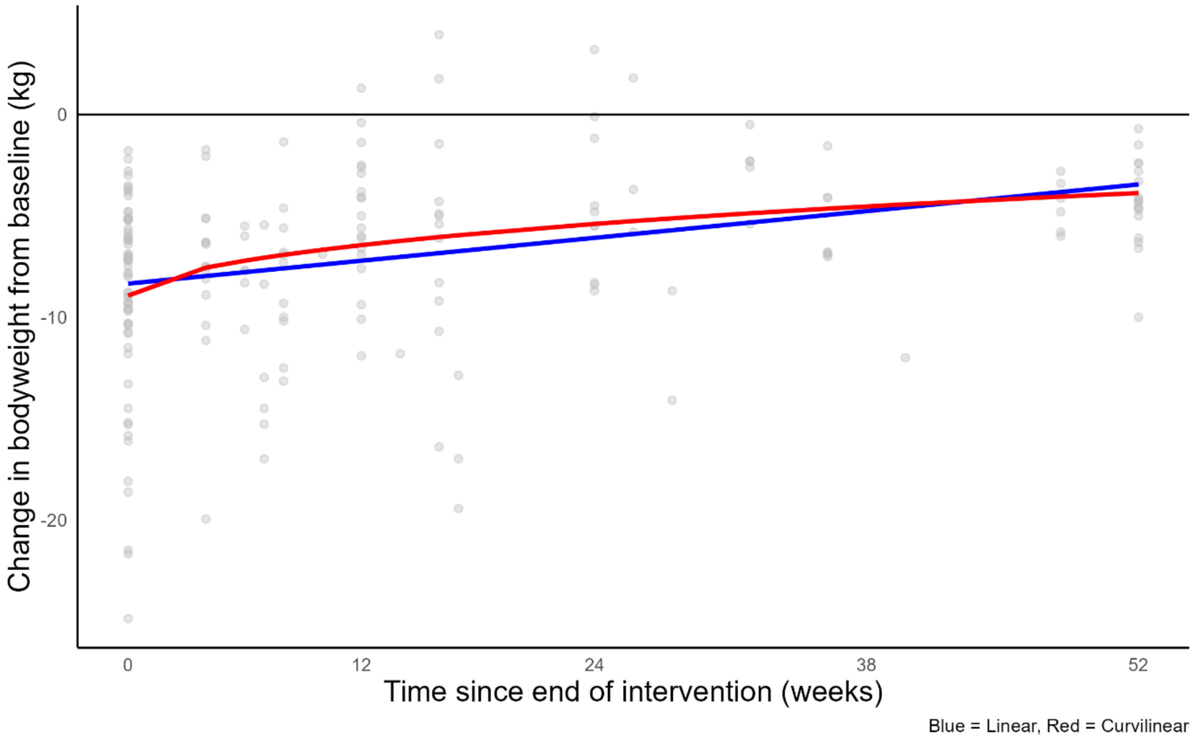

Supplementary Figure 9. Comparison of linear (blue) and curvilinear (red) models to assess the rate of weight regain after cessation of incretin mimetics.

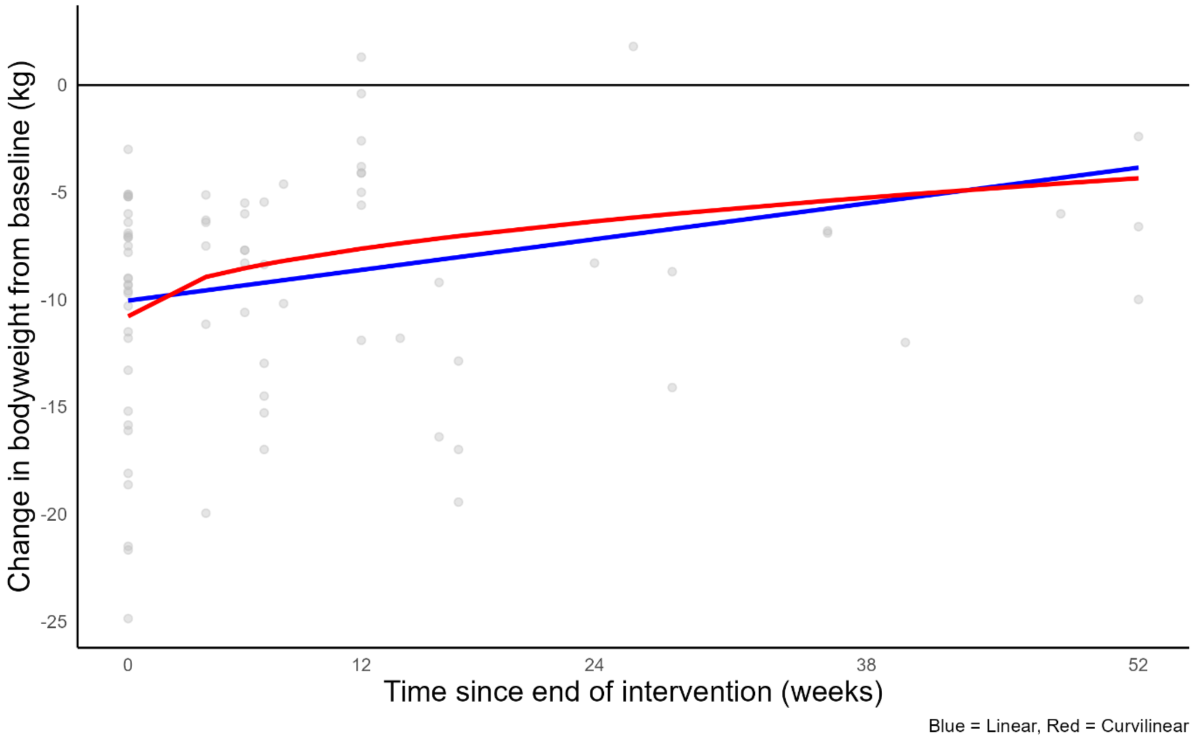

Supplementary Figure 10. Comparison of linear (blue) and curvilinear (red) models to assess the rate of weight regain after cessation of newer and more effective incretin mimetics.

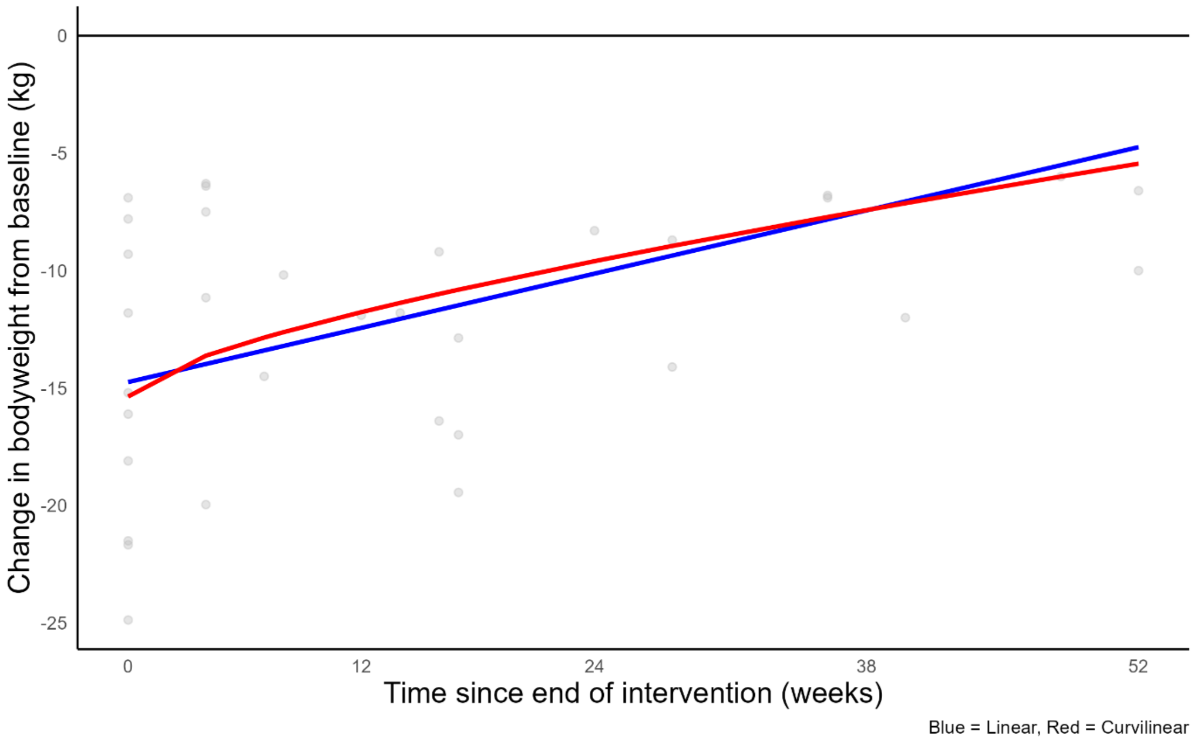

Supplementary Figure 11. Forest plot of all timepoints from studies using WMM included in the mixed model (Figure 1a). Data are plotted as weight change (kg) from baseline.

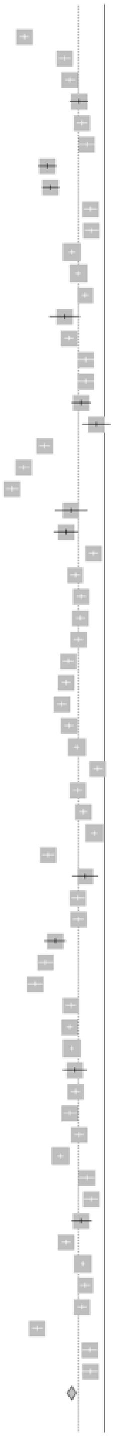

Study	N	Medication	Mean	95% CIs	Weight
Timepoint = Week 0					
Aronne 2023	335	Tirzepatide	-21.50	[-22.67; -20.33]	0.5%
Brownell 1981	69	Fenfluramine	-10.80	[-12.36; -9.24]	0.5%
Craighead 1984a	15	Fenfluramine	-9.30	[-10.57; -8.03]	0.5%
Craighead 1984b	14	Fenfluramine	-6.90	[-9.31; -4.49]	0.5%
Craighead 1984c	13	Fenfluramine	-6.00	[-7.36; -4.64]	0.5%
Craighead 1984d	16	Fenfluramine	-4.80	[-6.91; -2.69]	0.5%
Craighead 1981a	26	Fenfluramine	-15.30	[-17.64; -12.96]	0.5%
Craighead 1981b	33	Fenfluramine	-14.50	[-16.65; -12.35]	0.5%
Croghan 2016a	14	Lorcaserin	-3.70	[-5.43; -1.97]	0.5%
Croghan 2016b	15	Lorcaserin	-3.50	[-5.32; -1.68]	0.5%
Davidson 1999	141	Orlistat	-8.76	[-9.49; -8.03]	0.6%
Davies 2015a	317	Liraglutide	-7.10	[-7.78; -6.42]	0.6%
Davies 2015b	157	Liraglutide	-5.20	[-6.11; -4.29]	0.5%
Dawson 2011	8	Rimonabant	-10.73	[-14.77; -6.69]	0.5%
Early 2007	49	Sibutramine	-9.60	[-10.75; -8.45]	0.5%
Ferjan 2017a	12	Liraglutide	-5.10	[-7.14; -3.06]	0.5%
Ferjan 2017b	12	Liraglutide	-5.10	[-7.14; -3.06]	0.5%
Grilo 2014a	22	Sibutramine	-6.20	[-8.71; -3.69]	0.5%
Grilo 2014b	20	Sibutramine	-2.20	[-5.81; 1.41]	0.5%
Jastreboff 2024a	172	Tirzepatide	-16.11	[-17.45; -14.76]	0.5%
Jastreboff 2024b	185	Tirzepatide	-21.67	[-23.07; -20.27]	0.5%
Jastreboff 2024c	184	Tirzepatide	-24.87	[-26.37; -23.37]	0.5%
Jensterle 2024	25	Semaglutide*	-9.00	[-13.21; -4.79]	0.5%
Karhunen 2000	17	Orlistat	-10.40	[-13.63; -7.17]	0.5%
Khoo 2019	15	Liraglutide	-3.00	[-4.11; -1.89]	0.5%
Kwon 2022a	24	Orlistat + Phentermine	-7.90	[-9.18; -6.62]	0.5%
Kwon 2022b	27	Phentermine	-6.20	[-7.33; -5.07]	0.5%
Lau 2021a	101	Cagrilintide	-6.40	[-7.58; -5.22]	0.5%
Lau 2021b	100	Cagrilintide	-7.10	[-8.28; -5.92]	0.5%
Lau 2021c	102	Cagrilintide	-9.70	[-11.07; -8.33]	0.5%
Lau 2021d	102	Cagrilintide	-10.30	[-11.48; -9.12]	0.5%
Lau 2021e	101	Cagrilintide	-11.50	[-12.68; -10.32]	0.5%
Lau 2021f	99	Liraglutide	-9.60	[-10.78; -8.42]	0.5%
leRoux 2017	783	Liraglutide	-7.50	[-8.15; -6.85]	0.6%
Liang 2014	9	Topiramate	-1.79	[-3.03; -0.55]	0.5%
Marbury 1996a	54	Dexfenfluramine	-7.23	[-8.40; -6.06]	0.5%
Marbury 1996b	57	Dexfenfluramine	-5.63	[-6.62; -4.64]	0.5%
Marbury 1996c	59	Dexfenfluramine	-2.79	[-3.58; -2.00]	0.6%
McGowan 2024	138	Semaglutide	-15.20	[-16.77; -13.63]	0.5%
Moolla 2025	15	Liraglutide	-5.20	[-8.54; -1.86]	0.5%
Napolitano 2012	19	Sibutramine	-7.20	[-8.82; -5.58]	0.5%
Oneil 2018a	92	Semaglutide*	-7.01	[-8.35; -5.67]	0.5%
Oneil 2018b	96	Semaglutide*	-13.30	[-16.08; -10.52]	0.5%
Oneil 2018c	100	Semaglutide*	-15.85	[-17.61; -14.08]	0.5%
Oneil 2018d	100	Semaglutide*	-18.63	[-20.55; -16.71]	0.5%
Oneil 2018e	96	Liraglutide	-9.02	[-10.30; -7.75]	0.5%
Pi-Sunyer 2015	350	Liraglutide	-9.34	[-10.18; -8.50]	0.5%
Pi-Sunyer 2006	323	Rimonabant	-8.80	[-9.25; -8.35]	0.6%
Rodin 1988	16	DH	-8.00	[-11.14; -4.86]	0.5%
Rosenstock 2023b	90	Tirzepatide	-7.80	[-8.89; -6.71]	0.5%
Rosenstock 2023c	78	Tirzepatide	-9.30	[-10.90; -7.70]	0.5%
Rosenstock 2023a	98	Tirzepatide	-6.90	[-8.03; -5.77]	0.5%
Rubino 2021	268	Semaglutide	-11.80	[-12.57; -11.03]	0.6%
Samp 2015a	18	Orlistat	-4.79	[-6.83; -2.75]	0.5%
Samp 2015b	18	Sibutramine	-3.61	[-5.47; -1.75]	0.5%
Sathyapalan 2009	10	Rimonabant	-6.20	[-8.80; -3.60]	0.5%
Sjostrom 1998	138	Orlistat	-10.30	[-11.47; -9.13]	0.5%
Smith 2010	266	Lorcaserin	-5.80	[-6.20; -5.40]	0.6%
Svensson 2019	47	Liraglutide	-5.20	[-6.52; -3.88]	0.5%
Wadden 2013	159	Liraglutide	-6.00	[-7.13; -4.87]	0.5%
Wilding 2022	228	Semaglutide	-18.10	[-19.48; -16.72]	0.5%
Woo 2007a	27	Orlistat	-4.00	[-5.96; -2.04]	0.5%
Woo 2007b	28	Orlistat	-3.80	[-5.80; -1.80]	0.5%
Random effects model	6322		-8.84	[-10.08; -7.59]	33.6%

Heterogeneity: $I^2 = 98\%$, $\tau^2 = 23.8342$, $\chi^2_{62} = 3034.88$ ($p = 0$)

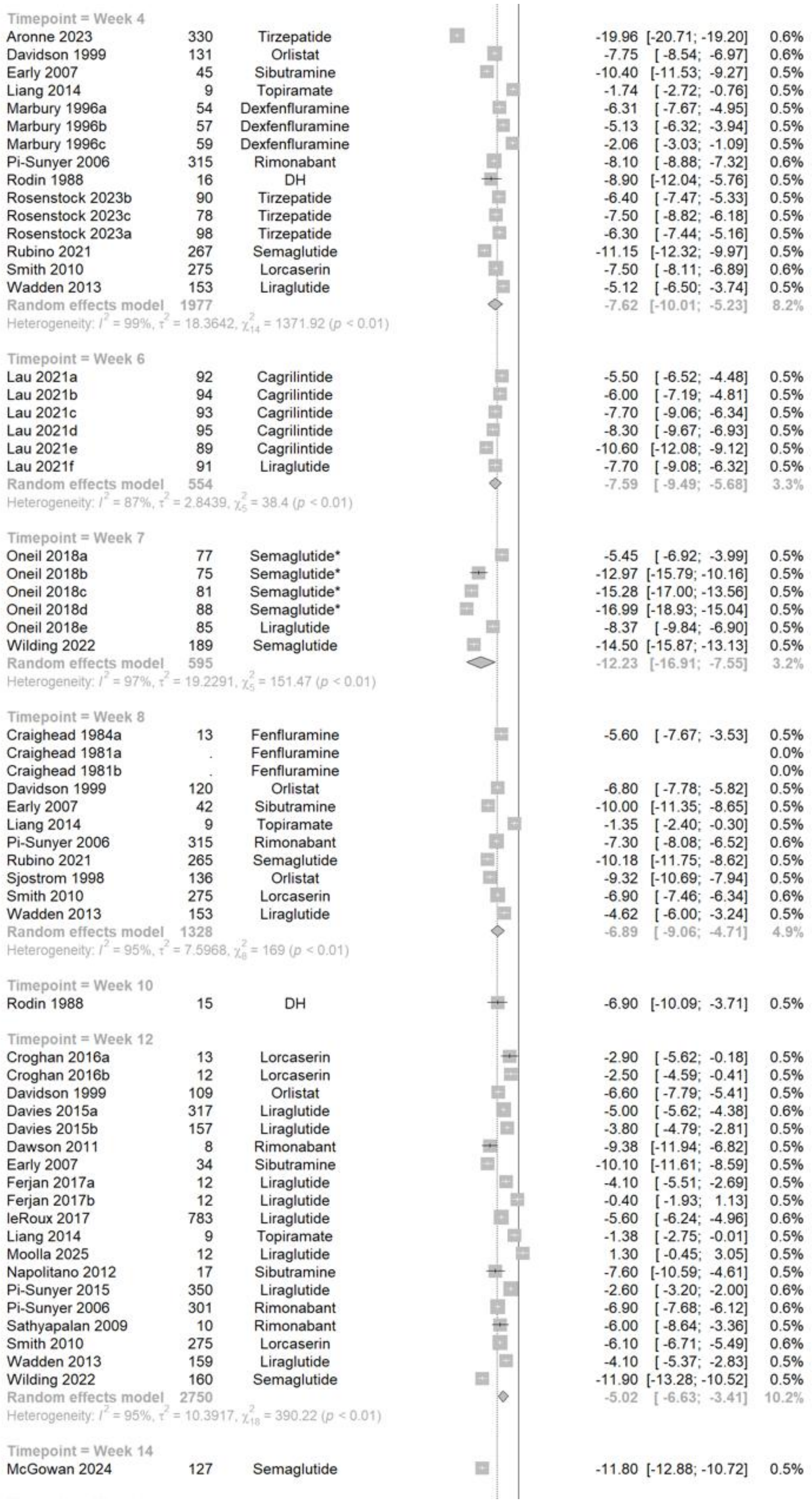

Timepoint = Week 4
Aronne 2023 330 Tirzepatide -19.96 [-20.71; -19.20] 0.6%
Davidson 1999 131 Orlistat -7.75 [-8.54; -6.97] 0.6%
Early 2007 45 Sibutramine -10.40 [-11.53; -9.27] 0.5%
Liang 2014 9 Topiramate -1.74 [-2.72; -0.76] 0.5%
Marbury 1996a 54 Dexfenfluramine -6.31 [-7.67; -4.95] 0.5%
Marbury 1996b 57 Dexfenfluramine -5.13 [-6.32; -3.94] 0.5%
Marbury 1996c 59 Dexfenfluramine -2.06 [-3.03; -1.09] 0.5%
Pi-Sunyer 2006 315 Rimonabant -8.10 [-8.88; -7.32] 0.6%
Rodin 1988 16 DH -8.90 [-12.04; -5.76] 0.5%
Rosenstock 2023b 90 Tirzepatide -6.40 [-7.47; -5.33] 0.5%
Rosenstock 2023c 78 Tirzepatide -7.50 [-8.82; -6.18] 0.5%
Rosenstock 2023a 98 Tirzepatide -6.30 [-7.44; -5.16] 0.5%
Rubino 2021 267 Semaglutide -11.15 [-12.32; -9.97] 0.5%
Smith 2010 275 Lorcaserin -7.50 [-8.11; -6.89] 0.6%
Wadden 2013 153 Liraglutide -5.12 [-6.50; -3.74] 0.5%
Random effects model 1977 -7.62 [-10.01; -5.23] 8.2%
Heterogeneity: I^2 = 99%, τ^2 = 18.3642, χ^2_{14} = 1371.92 (p < 0.01)
Timepoint = Week 6
Lau 2021a 92 Cagrilintide -5.50 [-6.52; -4.48] 0.5%
Lau 2021b 94 Cagrilintide -6.00 [-7.19; -4.81] 0.5%
Lau 2021c 93 Cagrilintide -7.70 [-9.06; -6.34] 0.5%
Lau 2021d 95 Cagrilintide -8.30 [-9.67; -6.93] 0.5%
Lau 2021e 89 Cagrilintide -10.60 [-12.08; -9.12] 0.5%
Lau 2021f 91 Liraglutide -7.70 [-9.08; -6.32] 0.5%
Random effects model 554 -7.59 [-9.49; -5.68] 3.3%
Heterogeneity: I^2 = 87%, τ^2 = 2.8439, χ^2_5 = 38.4 (p < 0.01)
Timepoint = Week 7
Oneil 2018a 77 Semaglutide* -5.45 [-6.92; -3.99] 0.5%
Oneil 2018b 75 Semaglutide* -12.97 [-15.79; -10.16] 0.5%
Oneil 2018c 81 Semaglutide* -15.28 [-17.00; -13.56] 0.5%
Oneil 2018d 88 Semaglutide* -16.99 [-18.93; -15.04] 0.5%
Oneil 2018e 85 Liraglutide -8.37 [-9.84; -6.90] 0.5%
Wilding 2022 189 Semaglutide -14.50 [-15.87; -13.13] 0.5%
Random effects model 595 -12.23 [-16.91; -7.55] 3.2%
Heterogeneity: I^2 = 97%, τ^2 = 19.2291, χ^2_5 = 151.47 (p < 0.01)
Timepoint = Week 8
Craighead 1984a 13 Fenfluramine -5.60 [-7.67; -3.53] 0.5%
Craighead 1981a . Fenfluramine 0.0%
Craighead 1981b . Fenfluramine 0.0%
Davidson 1999 120 Orlistat -6.80 [-7.78; -5.82] 0.5%
Early 2007 42 Sibutramine -10.00 [-11.35; -8.65] 0.5%
Liang 2014 9 Topiramate -1.35 [-2.40; -0.30] 0.5%
Pi-Sunyer 2006 315 Rimonabant -7.30 [-8.08; -6.52] 0.6%
Rubino 2021 265 Semaglutide -10.18 [-11.75; -8.62] 0.5%
Sjostrom 1998 136 Orlistat -9.32 [-10.69; -7.94] 0.5%
Smith 2010 275 Lorcaserin -6.90 [-7.46; -6.34] 0.6%
Wadden 2013 153 Liraglutide -4.62 [-6.00; -3.24] 0.5%
Random effects model 1328 -6.89 [-9.06; -4.71] 4.9%
Heterogeneity: I^2 = 95%, τ^2 = 7.5968, χ^2_8 = 169 (p < 0.01)
Timepoint = Week 10
Rodin 1988 15 DH -6.90 [-10.09; -3.71] 0.5%
Timepoint = Week 12
Croghan 2016a 13 Lorcaserin -2.90 [-5.62; -0.18] 0.5%
Croghan 2016b 12 Lorcaserin -2.50 [-4.59; -0.41] 0.5%
Davidson 1999 109 Orlistat -6.60 [-7.79; -5.41] 0.5%
Davies 2015a 317 Liraglutide -5.00 [-5.62; -4.38] 0.6%
Davies 2015b 157 Liraglutide -3.80 [-4.79; -2.81] 0.5%
Dawson 2011 8 Rimonabant -9.38 [-11.94; -6.82] 0.5%
Early 2007 34 Sibutramine -10.10 [-11.61; -8.59] 0.5%
Ferjan 2017a 12 Liraglutide -4.10 [-5.51; -2.69] 0.5%
Ferjan 2017b 12 Liraglutide -0.40 [-1.93; 1.13] 0.5%
leRoux 2017 783 Liraglutide -5.60 [-6.24; -4.96] 0.6%
Liang 2014 9 Topiramate -1.38 [-2.75; -0.01] 0.5%
Moolla 2025 12 Liraglutide 1.30 [-0.45; 3.05] 0.5%
Napolitano 2012 17 Sibutramine -7.60 [-10.59; -4.61] 0.5%
Pi-Sunyer 2015 350 Liraglutide -2.60 [-3.20; -2.00] 0.6%
Pi-Sunyer 2006 301 Rimonabant -6.90 [-7.68; -6.12] 0.6%
Sathyapalan 2009 10 Rimonabant -6.00 [-8.64; -3.36] 0.5%
Smith 2010 275 Lorcaserin -6.10 [-6.71; -5.49] 0.6%
Wadden 2013 159 Liraglutide -4.10 [-5.37; -2.83] 0.5%
Wilding 2022 160 Semaglutide -11.90 [-13.28; -10.52] 0.5%
Random effects model 2750 -5.02 [-6.63; -3.41] 10.2%
Heterogeneity: I^2 = 95%, τ^2 = 10.3917, χ^2_{18} = 390.22 (p < 0.01)
Timepoint = Week 14
McGowan 2024 127 Semaglutide -11.80 [-12.88; -10.72] 0.5%

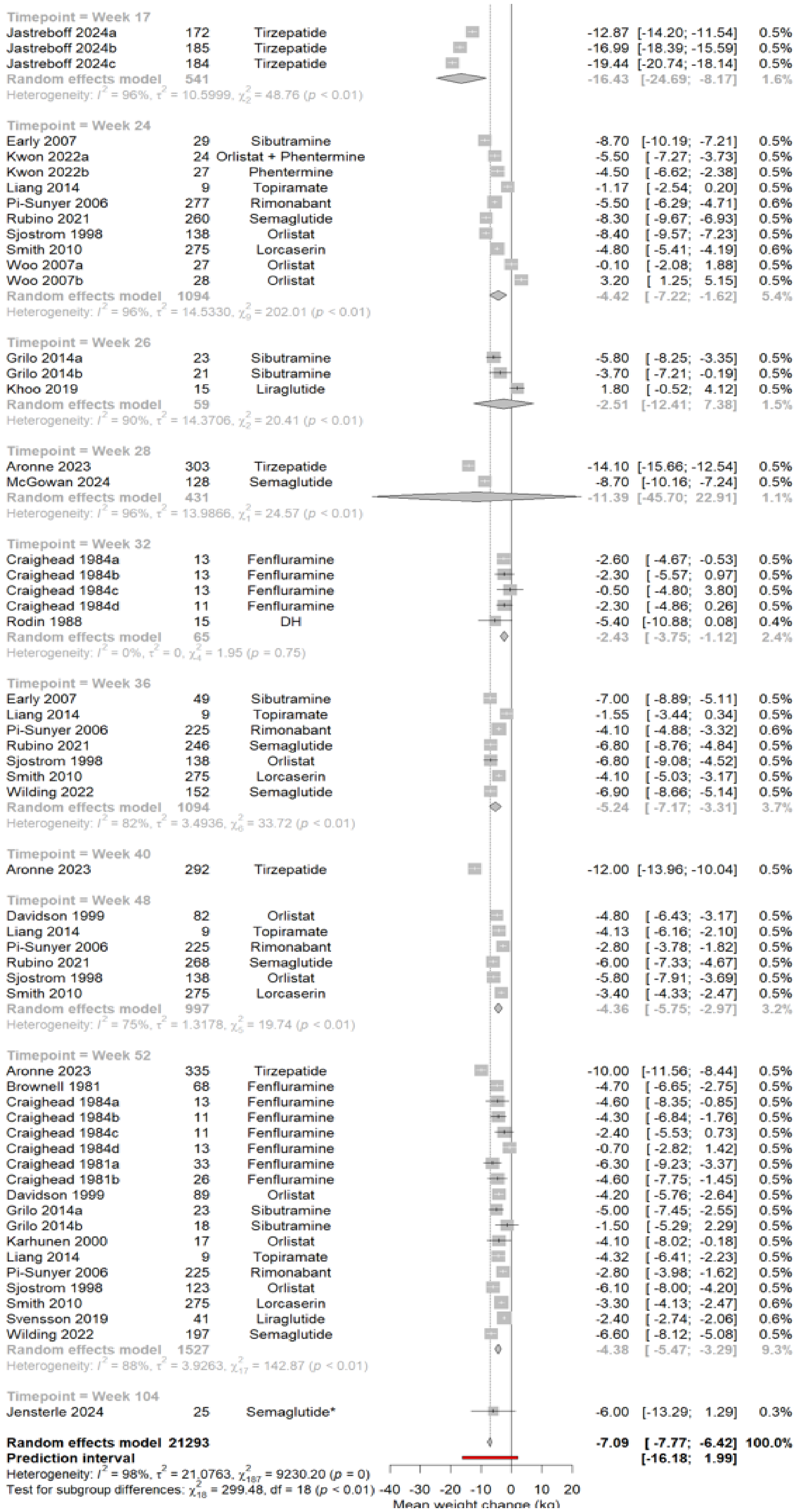

*Semaglutide indicates where dose below that recommended for weight management have been used.

Supplementary Figure 12. Forest plot of all timepoints from studies using incretin mimetic therapies included in the mixed model (Figure 1b). Data are plotted as weight change (kg) from baseline.

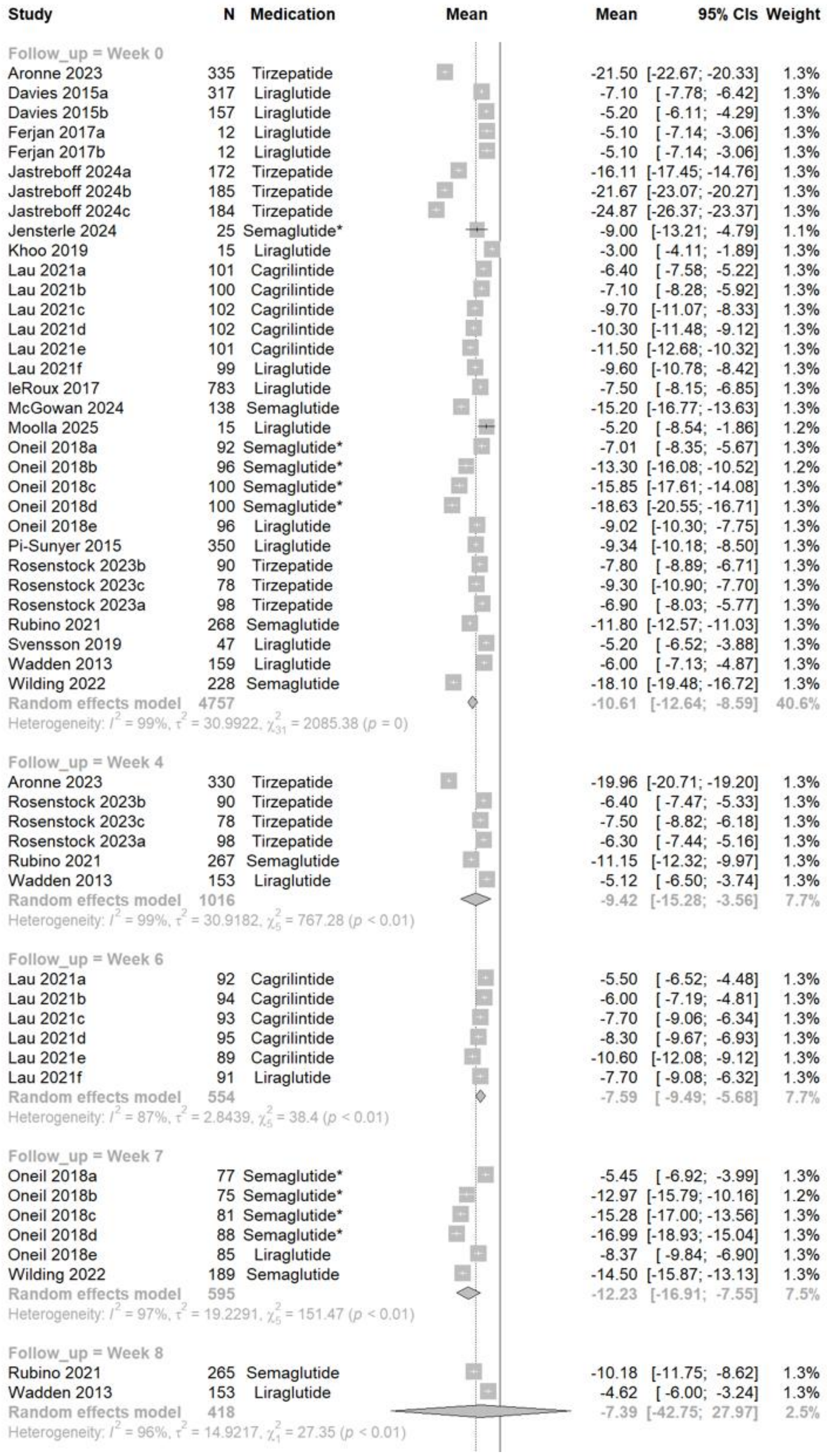

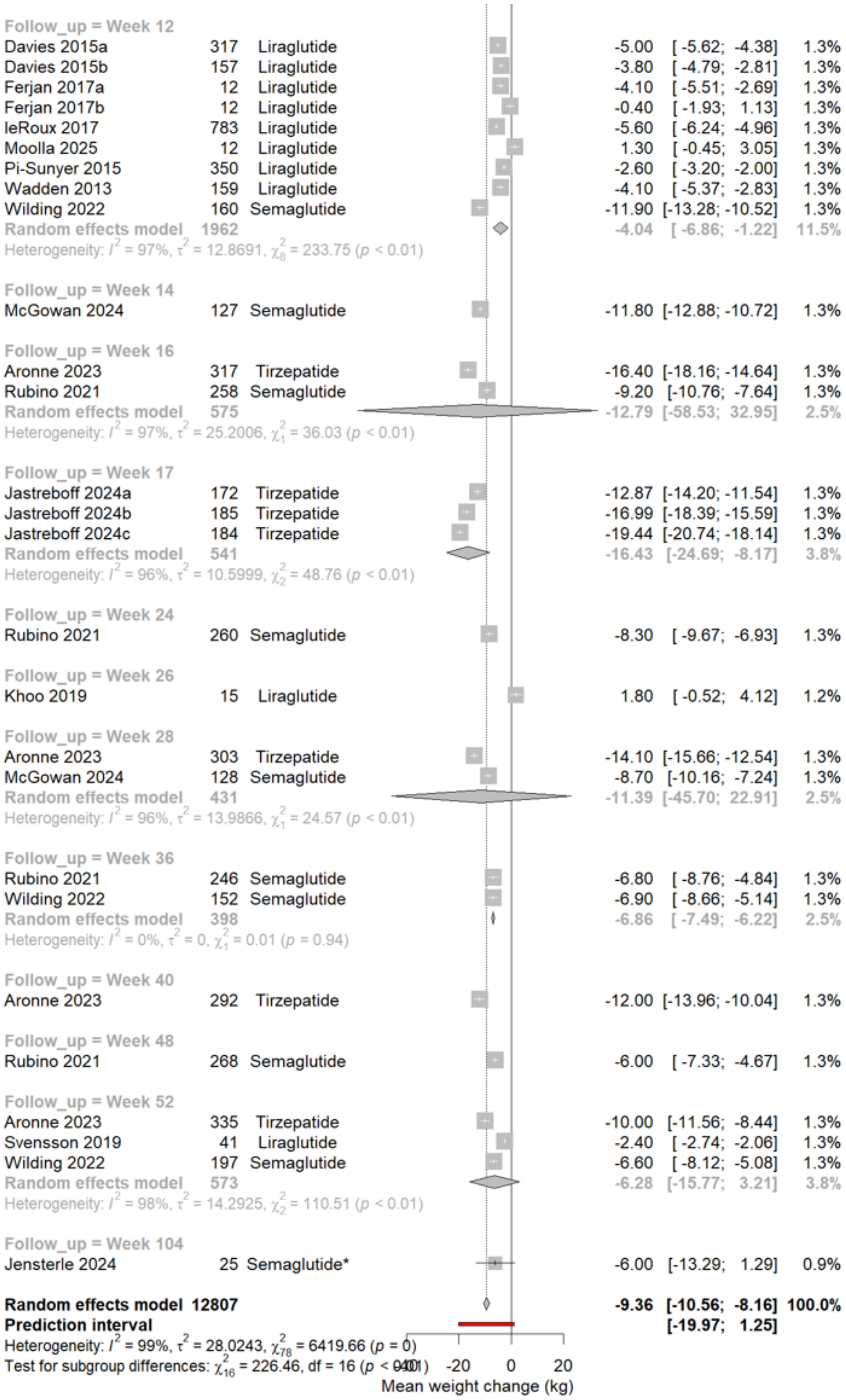

*Semaglutide indicates where dose below that recommended for weight management have been used.

Supplementary Figure 13. Forest plot of all timepoints from studies using newer and more effective incretin mimetic therapies included in the mixed model (Figure 1c). Data are plotted as weight change (kg) from baseline.

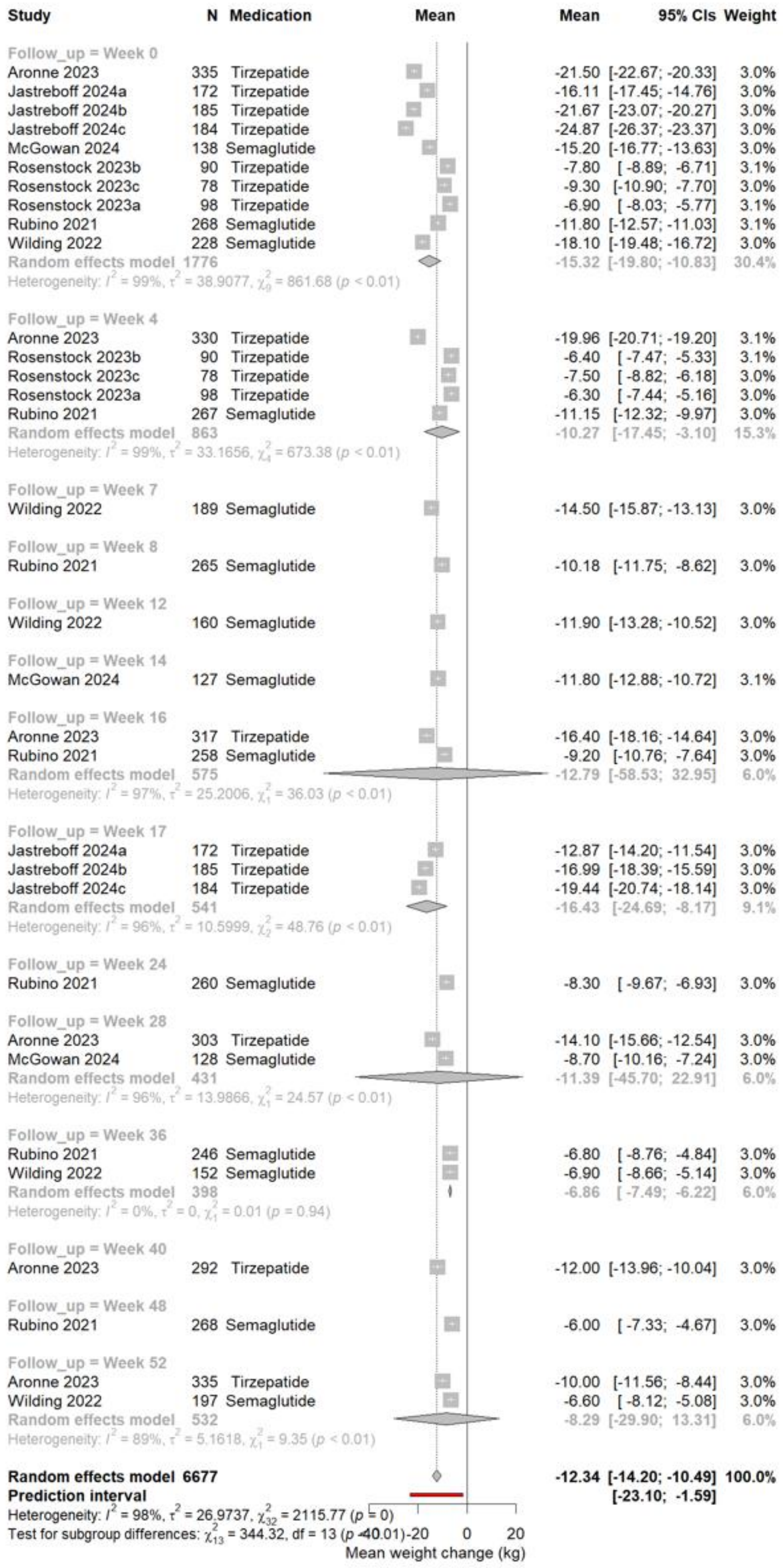

Supplementary Figure 14. Forest plot of all timepoints from RCTs using WMM included in the mixed model (Figure 2a). Data are plotted as difference in weight change (kg) from baseline between intervention and control.

Study	Intervention (n)	Comparator (n)	Medication	Mean Difference	MD	95% CIs	Weight
Follow_up = Week 0							
Brownell 1981	69	43	Fenfluramine		-3.70	[-5.78; -1.62]	0.8%
Craighead 1984a	15	4	Fenfluramine		-3.60	[-7.44; 0.24]	0.6%
Craighead 1984b	14	4	Fenfluramine		-1.20	[-5.55; 3.15]	0.6%
Craighead 1984c	13	4	Fenfluramine		-0.30	[-4.17; 3.57]	0.6%
Craighead 1984d	16	4	Fenfluramine		0.90	[-3.29; 5.09]	0.6%
Craighead 1981a	26	17	Fenfluramine		-4.40	[-7.98; -0.82]	0.6%
Craighead 1981b	33	17	Fenfluramine		-3.60	[-7.06; -0.14]	0.6%
Croghan 2016a	14	8	Lorcaserin		-2.70	[-6.20; 0.80]	0.6%
Croghan 2016b	15	8	Lorcaserin		-2.50	[-6.05; 1.05]	0.6%
Davidson 1999	141	133	Orlistat		-2.95	[-4.45; -1.45]	0.8%
Davies 2015a	317	58	Liraglutide		-4.00	[-5.66; -2.34]	0.8%
Davies 2015b	157	58	Liraglutide		-2.10	[-3.87; -0.33]	0.8%
Dawson 2011	8	8	Rimonabant		-12.33	[-18.00; -6.66]	0.5%
Grilo 2014a	22	11	Sibutramine		-7.20	[-10.92; -3.48]	0.6%
Grilo 2014b	20	11	Sibutramine		-3.20	[-7.73; 1.33]	0.6%
Jastreboff 2024a	172	131	Tirzepatide		-12.90	[-15.15; -10.65]	0.7%
Jastreboff 2024b	185	131	Tirzepatide		-18.46	[-20.82; -16.10]	0.7%
Jastreboff 2024c	184	131	Tirzepatide		-21.66	[-24.00; -19.32]	0.7%
Karhunen 2000	17	19	Orlistat		-1.30	[-5.45; 2.85]	0.6%
Khoo 2019	15	15	Liraglutide		0.50	[-1.51; 2.51]	0.8%
Lau 2021a	101	101	Cagrilintide		-3.10	[-4.76; -1.44]	0.8%
Lau 2021b	100	101	Cagrilintide		-3.80	[-5.46; -2.14]	0.8%
Lau 2021c	102	101	Cagrilintide		-6.40	[-8.21; -4.59]	0.8%
Lau 2021d	102	101	Cagrilintide		-7.00	[-8.66; -5.34]	0.8%
Lau 2021e	101	101	Cagrilintide		-8.20	[-9.86; -6.54]	0.8%
Lau 2021f	99	101	Liraglutide		-6.30	[-7.96; -4.64]	0.8%
leRoux 2017	783	326	Liraglutide		-4.70	[-5.82; -3.58]	0.8%
Marbury 1996a	54	18	Dexfenfluramine		-4.40	[-6.18; -2.62]	0.8%
Marbury 1996b	57	18	Dexfenfluramine		-2.80	[-4.46; -1.14]	0.8%
Marbury 1996c	59	18	Dexfenfluramine		0.04	[-1.52; 1.60]	0.8%
McGowan 2024	138	69	Semaglutide		-12.40	[-14.36; -10.44]	0.8%
Moolla 2025	15	14	Liraglutide		-0.50	[-4.36; 3.36]	0.6%
Napolitano 2012	19	19	Sibutramine		-4.40	[-6.30; -2.50]	0.8%
Oneil 2018a	92	123	Semaglutide*		-4.39	[-6.12; -2.65]	0.8%
Oneil 2018b	96	123	Semaglutide*		-10.67	[-13.73; -7.61]	0.7%
Oneil 2018c	100	123	Semaglutide*		-13.22	[-15.30; -11.14]	0.8%
Oneil 2018d	100	123	Semaglutide*		-16.01	[-18.22; -13.79]	0.8%
Oneil 2018e	96	123	Liraglutide		-6.40	[-8.08; -4.71]	0.8%
Pi-Sunyer 2015	350	304	Liraglutide		-13.05	[-14.30; -11.80]	0.8%
Pi-Sunyer 2006	323	292	Rimonabant		-6.00	[-6.57; -5.43]	0.8%
Rodin 1988	16	16	DH		-1.50	[-6.00; 3.00]	0.6%
Rosenstock 2023b	90	57	Tirzepatide		-6.70	[-7.99; -5.41]	0.8%
Rosenstock 2023c	78	57	Tirzepatide		-8.20	[-9.94; -6.46]	0.8%
Rosenstock 2023a	98	57	Tirzepatide		-5.80	[-7.12; -4.48]	0.8%
Sathyapalan 2009	10	10	Rimonabant		-4.60	[-7.87; -1.33]	0.7%
Sjostrom 1998	138	123	Orlistat		-4.20	[-5.72; -2.68]	0.8%
Smith 2010	266	665	Lorcaserin		-3.60	[-4.04; -3.16]	0.8%
Svensson 2019	47	50	Liraglutide		-5.70	[-7.91; -3.49]	0.8%
Wadden 2013	159	144	Liraglutide		-5.90	[-7.50; -4.30]	0.8%
Wilding 2022	228	99	Semaglutide		-15.90	[-18.24; -13.56]	0.7%
Random effects model	5470	4392			-6.14	[-7.24; -5.04]	36.3%
Heterogeneity: $I^2 = 95\%$, $\tau^2 = 14.1143$, $\chi^2_{49} = 1051.08$ ($p < 0.01$)							
Follow_up = Week 4							
Davidson 1999	131	118	Orlistat		-2.12	[-3.23; -1.01]	0.8%
Marbury 1996a	54	18	Dexfenfluramine		-3.74	[-5.65; -1.83]	0.8%
Marbury 1996b	57	18	Dexfenfluramine		-2.56	[-4.35; -0.77]	0.8%
Marbury 1996c	59	18	Dexfenfluramine		0.51	[-1.14; 2.16]	0.8%
Pi-Sunyer 2006	315	284	Rimonabant		-5.20	[-6.31; -4.09]	0.8%
Rodin 1988	16	16	DH		-1.60	[-6.12; 2.92]	0.6%
Rosenstock 2023b	90	57	Tirzepatide		-5.40	[-6.70; -4.10]	0.8%
Rosenstock 2023c	78	57	Tirzepatide		-6.50	[-8.01; -4.99]	0.8%
Rosenstock 2023a	98	57	Tirzepatide		-5.30	[-6.66; -3.94]	0.8%
Smith 2010	275	684	Lorcaserin		-4.20	[-4.91; -3.49]	0.8%
Wadden 2013	153	141	Liraglutide		-5.12	[-6.93; -3.31]	0.8%
Random effects model	1326	1468			-3.90	[-5.00; -2.80]	8.6%
Heterogeneity: $I^2 = 85\%$, $\tau^2 = 2.7478$, $\chi^2_{10} = 67.9$ ($p < 0.01$)							
Follow_up = Week 6							
Lau 2021a	92	84	Cagrilintide		-2.60	[-4.31; -0.89]	0.8%
Lau 2021b	94	84	Cagrilintide		-3.10	[-4.92; -1.28]	0.8%
Lau 2021c	93	84	Cagrilintide		-4.80	[-6.73; -2.87]	0.8%
Lau 2021d	95	84	Cagrilintide		-5.40	[-7.33; -3.47]	0.8%
Lau 2021e	89	84	Cagrilintide		-7.70	[-9.71; -5.69]	0.8%
Lau 2021f	91	84	Liraglutide		-4.80	[-6.74; -2.86]	0.8%
Random effects model	554	504			-4.69	[-6.14; -3.24]	4.6%
Heterogeneity: $I^2 = 72\%$, $\tau^2 = 2.3552$, $\chi^2_{5} = 17.74$ ($p < 0.01$)							
Follow_up = Week 7							
Oneil 2018a	77	102	Semaglutide*		-3.40	[-5.35; -1.44]	0.8%
Oneil 2018b	75	102	Semaglutide*		-10.92	[-14.08; -7.75]	0.7%
Oneil 2018c	81	102	Semaglutide*		-13.23	[-15.38; -11.07]	0.8%
Oneil 2018d	88	102	Semaglutide*		-14.93	[-17.27; -12.60]	0.7%
Oneil 2018e	85	102	Liraglutide		-6.31	[-8.27; -4.36]	0.8%
Wilding 2022	189	88	Semaglutide		-12.20	[-14.28; -10.12]	0.8%
Random effects model	595	598			-10.13	[-13.90; -6.37]	4.5%
Heterogeneity: $I^2 = 94\%$, $\tau^2 = 20.7586$, $\chi^2_{5} = 87.02$ ($p < 0.01$)							

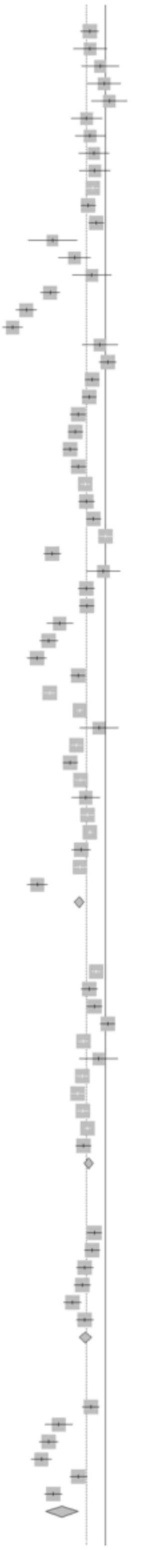

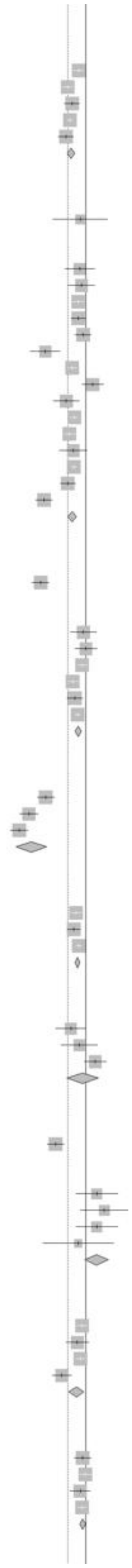

Follow_up = Week 8						
Craighead 1981a	.	.	Fenfluramine			0.0%
Craighead 1981b	.	.	Fenfluramine			0.0%
Davidson 1999	120	110	Orlistat	-1.80	[-3.06; -0.54]	0.8%
Pi-Sunyer 2006	315	284	Rimonabant	-4.50	[-5.75; -3.25]	0.8%
Sjostrom 1998	136	126	Orlistat	-3.43	[-5.23; -1.62]	0.8%
Smith 2010	275	684	Lorcaserin	-3.90	[-4.53; -3.27]	0.8%
Wadden 2013	153	141	Liraglutide	-4.91	[-6.72; -3.11]	0.8%
Random effects model	999	1345		-3.67	[-4.66; -2.68]	4.0%
Heterogeneity: $I^2 = 68\%$, $\tau^2 = 0.8163$, $\chi^2_4 = 12.56$ ($p = 0.01$)						
Follow_up = Week 10						
Rodin 1988	15	15	DH	-1.40	[-8.10; 5.30]	0.4%
Follow_up = Week 12						
Croghan 2016a	13	8	Lorcaserin	-1.50	[-5.19; 2.19]	0.6%
Croghan 2016b	12	8	Lorcaserin	-1.10	[-4.36; 2.16]	0.7%
Davidson 1999	109	99	Orlistat	-1.90	[-3.44; -0.36]	0.8%
Davies 2015a	317	58	Liraglutide	-1.90	[-3.71; -0.09]	0.8%
Davies 2015b	157	58	Liraglutide	-0.70	[-2.66; 1.26]	0.8%
Dawson 2011	8	8	Rimonabant	-9.98	[-13.63; -6.33]	0.6%
leRoux 2017	783	326	Liraglutide	-3.40	[-4.52; -2.28]	0.8%
Moolla 2025	12	12	Liraglutide	1.70	[-0.95; 4.35]	0.7%
Napolitano 2012	17	17	Sibutramine	-4.80	[-7.97; -1.63]	0.7%
Pi-Sunyer 2015	350	304	Liraglutide	-2.90	[-3.74; -2.06]	0.8%
Pi-Sunyer 2006	301	254	Rimonabant	-4.00	[-5.26; -2.74]	0.8%
Sathyapalan 2009	10	10	Rimonabant	-3.10	[-6.43; 0.23]	0.7%
Smith 2010	275	684	Lorcaserin	-3.00	[-3.71; -2.29]	0.8%
Wadden 2013	159	141	Liraglutide	-4.40	[-6.20; -2.60]	0.8%
Wilding 2022	160	75	Semaglutide	-10.30	[-12.38; -8.22]	0.8%
Random effects model	2683	2062		-3.35	[-4.44; -2.27]	11.2%
Heterogeneity: $I^2 = 84\%$, $\tau^2 = 3.3615$, $\chi^2_{14} = 89.6$ ($p < 0.01$)						
Follow_up = Week 14						
McGowan 2024	127	63	Semaglutide	-11.10	[-13.20; -9.00]	0.8%
Follow_up = Week 16						
Craighead 1984a	13	5	Fenfluramine	-0.60	[-3.81; 2.61]	0.7%
Craighead 1984d	13	4	Fenfluramine	0.00	[-2.74; 2.74]	0.7%
Davidson 1999	107	98	Orlistat	-0.90	[-2.20; 0.40]	0.8%
Pi-Sunyer 2006	301	254	Rimonabant	-3.20	[-4.46; -1.94]	0.8%
Sjostrom 1998	138	126	Orlistat	-2.70	[-4.65; -0.75]	0.8%
Smith 2010	275	684	Lorcaserin	-2.00	[-2.68; -1.32]	0.8%
Random effects model	847	1171		-1.88	[-2.73; -1.03]	4.6%
Heterogeneity: $I^2 = 48\%$, $\tau^2 = 0.4777$, $\chi^2_5 = 9.53$ ($p = 0.09$)						
Follow_up = Week 17						
Jastreboff 2024a	172	136	Tirzepatide	-9.86	[-11.97; -7.75]	0.8%
Jastreboff 2024b	185	136	Tirzepatide	-13.98	[-16.22; -11.75]	0.7%
Jastreboff 2024c	184	136	Tirzepatide	-16.43	[-18.61; -14.26]	0.8%
Random effects model	541	408		-13.42	[-17.24; -9.60]	2.3%
Heterogeneity: $I^2 = 89\%$, $\tau^2 = 10.1620$, $\chi^2_2 = 18.5$ ($p < 0.01$)						
Follow_up = Week 24						
Pi-Sunyer 2006	277	247	Rimonabant	-2.40	[-3.66; -1.14]	0.8%
Sjostrom 1998	138	126	Orlistat	-3.00	[-4.66; -1.34]	0.8%
Smith 2010	275	684	Lorcaserin	-1.80	[-2.49; -1.11]	0.8%
Random effects model	690	1057		-2.08	[-2.66; -1.49]	2.4%
Heterogeneity: $I^2 = 3\%$, $\tau^2 = 0.0097$, $\chi^2_2 = 2.06$ ($p = 0.36$)						
Follow_up = Week 26						
Grilo 2014a	23	11	Sibutramine	-3.70	[-7.41; 0.01]	0.6%
Grilo 2014b	21	11	Sibutramine	-1.60	[-6.07; 2.87]	0.6%
Khoo 2019	15	15	Liraglutide	2.30	[-0.42; 5.02]	0.7%
Random effects model	59	37		-0.80	[-4.69; 3.08]	1.9%
Heterogeneity: $I^2 = 72\%$, $\tau^2 = 8.3780$, $\chi^2_2 = 7.05$ ($p = 0.03$)						
Follow_up = Week 28						
McGowan 2024	128	69	Semaglutide	-7.50	[-9.53; -5.47]	0.8%
Follow_up = Week 32						
Craighead 1984b	13	5	Fenfluramine	2.70	[-2.46; 7.86]	0.5%
Craighead 1984c	13	5	Fenfluramine	4.50	[-1.37; 10.37]	0.5%
Craighead 1984d	11	4	Fenfluramine	2.70	[-2.45; 7.85]	0.5%
Rodin 1988	15	15	DH	-1.90	[-10.54; 6.74]	0.3%
Random effects model	52	29		2.62	[-0.29; 5.54]	1.8%
Heterogeneity: $I^2 = 0\%$, $\tau^2 = 0$, $\chi^2_3 = 1.45$ ($p = 0.69$)						
Follow_up = Week 36						
Pi-Sunyer 2006	225	216	Rimonabant	-1.00	[-2.25; 0.25]	0.8%
Sjostrom 1998	138	126	Orlistat	-2.10	[-4.85; 0.65]	0.7%
Smith 2010	275	684	Lorcaserin	-1.30	[-2.36; -0.24]	0.8%
Wilding 2022	152	75	Semaglutide	-5.90	[-8.26; -3.54]	0.7%
Random effects model	790	1101		-2.35	[-4.15; -0.55]	3.1%
Heterogeneity: $I^2 = 79\%$, $\tau^2 = 2.4768$, $\chi^2_3 = 14.03$ ($p < 0.01$)						
Follow_up = Week 48						
Davidson 1999	82	81	Orlistat	-0.80	[-2.80; 1.20]	0.8%
Pi-Sunyer 2006	225	216	Rimonabant	-0.10	[-1.63; 1.43]	0.8%
Sjostrom 1998	138	126	Orlistat	-1.40	[-3.93; 1.13]	0.7%
Smith 2010	275	684	Lorcaserin	-1.00	[-2.03; 0.03]	0.8%
Random effects model	720	1107		-0.79	[-1.54; -0.04]	3.1%
Heterogeneity: $I^2 = 0\%$, $\tau^2 = 0$, $\chi^2_3 = 1.17$ ($p = 0.76$)						

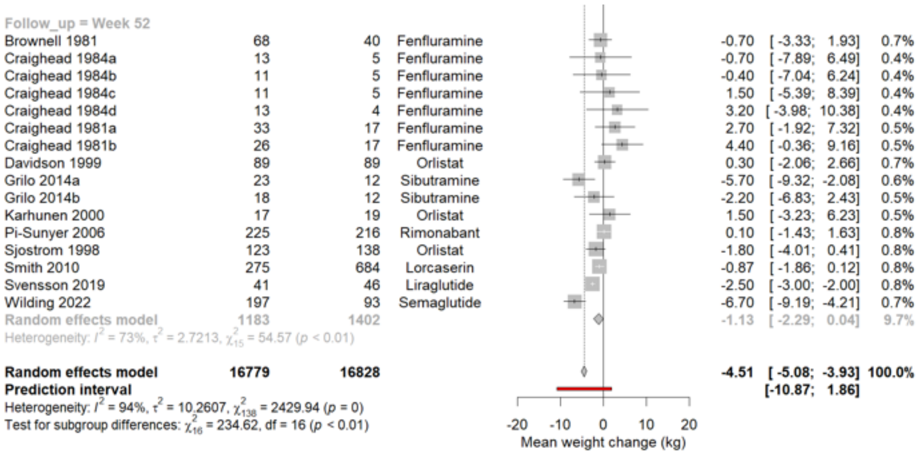

*Semaglutide indicates where dose below that recommended for weight management have been used.

Supplementary Figure 15. Forest plot of all timepoints from RCTs using incretin mimetic therapies included in the mixed model (Figure 2b). Data are plotted as difference in weight change (kg) from baseline between intervention and control.

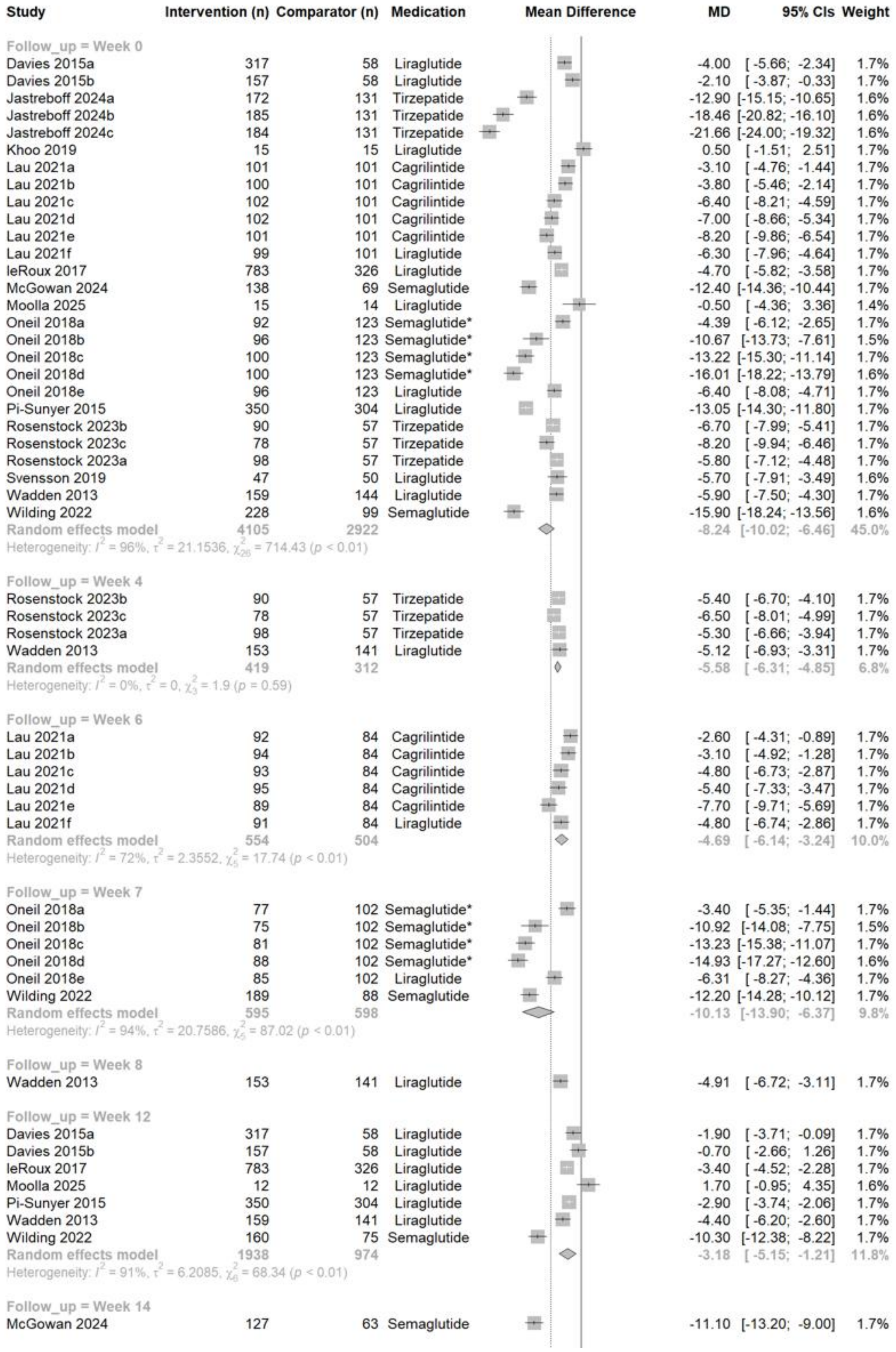

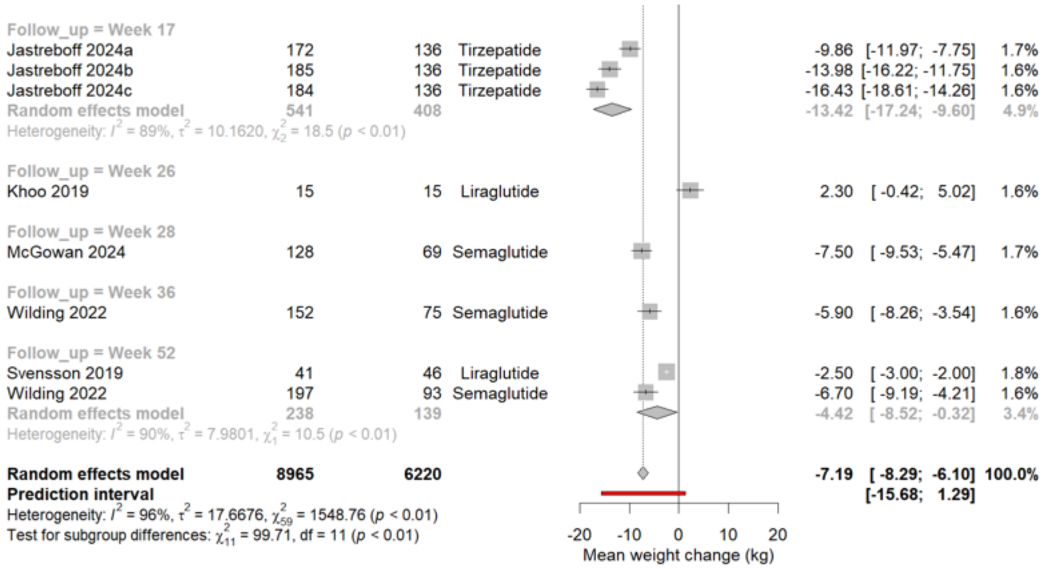

*Semaglutide indicates where dose below that recommended for weight management have been used.

Supplementary Figure 16. Forest plot of all timepoints from RCTs using newer and more effective incretin mimetic therapies included in the mixed model (Figure 2c). Data are plotted as difference in weight change (kg) from baseline between intervention and control.

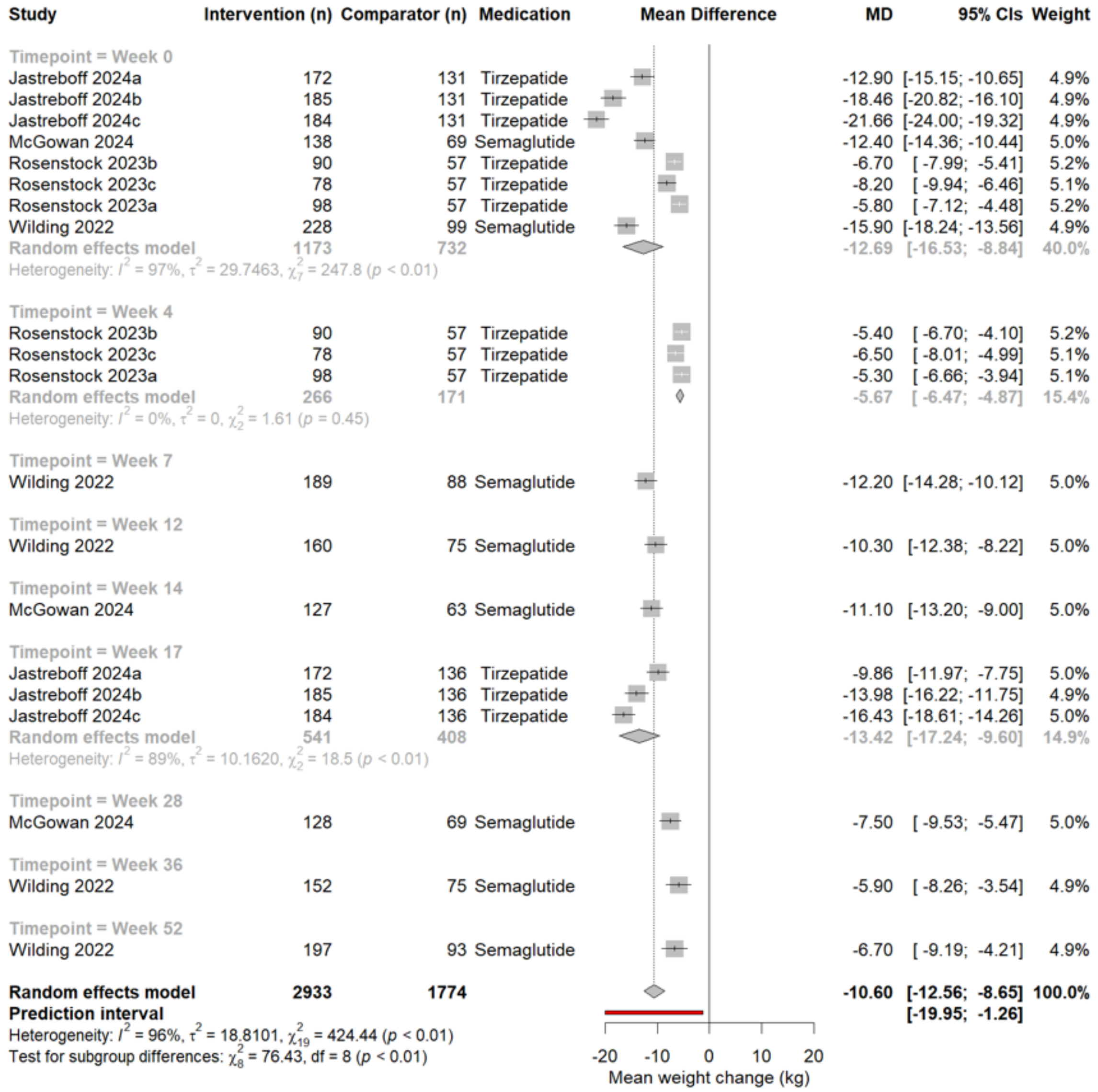

Supplement: Supplementary file 1 — Supplementary information: Additional tables 1-6 and figures 1-16 [file wess085304.ww.pdf]
